# Comprehensive pan-cancer analysis reveals CGB5 is a potential promising predictive and immunotherapeutic biomarker

**DOI:** 10.3389/fmed.2025.1624815

**Published:** 2025-09-19

**Authors:** Shuni Chen, Shan Lin, Feng Li, Haibin He, Yuanjie Zhang, Guiping Ma, Weifeng Yu

**Affiliations:** ^1^Shenzhen Hospital, Beijing University of Chinese Medicine, Shenzhen, Guangdong, China; ^2^Department of Gastroenterology, Shenzhen Hospital of Integrated Traditional and Western Medicine, Shenzhen, Guangdong, China

**Keywords:** pan-cancer, gastric cancer, prognosis, oncogene, biomarker

## Abstract

**Background:**

Existing studies substantiate the notion that CGB5 plays a pivotal role in various cancers, including gastric and ovarian cancers, and is strongly associated with patient prognosis. However, to date, there have been no comprehensive reports investigating the role of CGB5 in pan-cancer analysis.

**Methods:**

In this study, an in-depth investigation of CGB5 in pan-cancer was conducted through multiple public databases, including The Cancer Genome Atlas (TCGA), Human Protein Atlas (HPA), UALCAN, cBioPortal Platform, Gene Set Cancer Analysis (GSCA), Kaplan–Meier Plotter, TIMER, TISIDB, SangerBox Website, and Metascape database. The genomic, transcriptomic, epigenetic, immune microenvironmental, and clinical prognostic significance of CGB5 across various cancers was systematically analyzed. Furthermore, CGB5 expression in gastric cancer cells was experimentally detected, and the potential mechanisms underlying its impact on prognosis were elucidated.

**Results:**

This study shows that CGB5 exhibits diverse expression patterns in most tumors, including high, low, or no significant expression changes. Compared to normal tissues, CGB5 is significantly up-regulated in six tumor types, such as liver, lung, and gastric cancers. Its expression correlates positively with tumor stroma content and immune grading but negatively with immunological markers. Additionally, CGB5 is associated with specific immune sub-types in various cancers, including endometrial, testicular germ cell, and gastric adenocarcinoma, and closely linked to clinical features of gastric cancer patients. CGB5 primarily involves immune-related pathways, such as “Primary immunodeficiency,” “CD8 TCR signaling pathway,” and “PD-1 checkpoint signaling.”

**Conclusion:**

This study demonstrates that CGB5 expression is closely associated with immune cell infiltration across cancer types, showing significant variation in infiltration patterns among tumor types. CGB5 is significantly up-regulated in various malignancies and strongly correlates with cancer patient prognoses, specifically in malignancies like GC and PAAD. Overall, these findings indicate CGB5 as a promising biomarker for pan-cancer diagnosis and prognosis.

## 1 Introduction

Cancer is a global health challenge that significantly impacts human well being and quality of life. According to World Health Organization (WHO) statistics, cancer is currently the leading cause of mortality worldwide (1). Further research has indicated that among all types of cancer, lung, breast, and stomach cancers exhibit higher incidence and mortality rates (2). Despite substantial advancements in cancer treatments, such as d targeted therapies, immunotherapy, and radiotherapy, the overall survival (OS) rate within a 5-year period remains sub-optimal for many patients (3). Additionally, the economic burden imposed by cancer on society is substantial and cannot be overlooked (4). Consequently, the identification of effective cancer biomarkers holds critical significance in improving prognostic outcomes for individuals diagnosed with cancer and alleviating the societal burden.

The human chorionic gonadotropin (hCG) beta-subunit, a hormone-specific entity, is produced by Placental syncytiotrophoblasts. Its encoding is facilitated by four distinct chorionic gonadotropin β genes (CGB, CGB5, CGB7, and CGB8), which are located within the LHB/CGB gene cluster on chromosome 19q13.3 (5). Among these four duplicated hCG beta genes, CGB5 and CGB8 exhibit the highest transcriptional activity, accounting for 62%−82% of the total beta subunit mRNA transcript pool (6). The role of CGB5 in tumors has garnered significant attention. Previous studies have provided evidence to supporting the notion that CGB5 plays a critical role in gastric cancer (7, 8), ovarian cancer (9), and squamous lung cancer (10), and is closely associated with patient prognosis. However, to date, there have been no comprehensive reports investigating the role of CGB5 across pan-cancer.

In the present study, a comprehensive analysis of the genomics, transcriptomics, epigenetics, immune microenvironment, and clinical and prognostic significance of CGB5 was conducted across various cancer types. Additionally, experimental studies were performed to examine the expression level of CGB5 in gastric cancer and to elucidate the potential mechanisms underlying its impacts on prognosis.

## 2 Materials and methods

### 2.1 Data collection

Expression profiling and clinical data for 33 tumor types were retrieved from The Cancer Genome Atlas (TCGA) database (https://portal.gdc.cancer.gov/). Additionally, the TCGA_GTEx database, which integrates tumor samples from TCGA along with their matched normal tissue samples, was obtained from the Xena database provided by the University of California, Santa Cruz (UCSC; https://xenabrowser.net/datapages/). The immunohistochemistry images of healthy and malignant human tissues were sourced from the Human Protein Atlas (HPA; https://www.proteinatlas.org/). This study complied with the guidelines established by TCGA and UCSC, thereby eliminating the need for additional ethical approval or informed consent from patients.

### 2.2 Expression analysis

The mRNA expression levels of CGB5 were compared between normal and tumor tissues using TCGA samples as well as paired TCGA and TCGA_GTEx samples. Additionally, the protein expression levels of CGB5 in both normal and malignant tissues were analyzed using data from the Human Protein Atlas (HPA) and the UALCAN databases (https://ualcan.path.uab.edu/analysis.html) (11).

### 2.3 Genetic alteration test

The cBioPortal platform (https://www.cbioportal.org), as described in detail elsewhere (12), was utilized to collect data on variant frequencies, variant types, and variant sites in all TCGA tumors datasets. The UALCAN platform was employed to investigate the DNA methylation patterns of CGB5 across various tumor types. Gene Set Cancer Analysis (GSCA, https://github.com/zji90/GSCA) (13) is a comprehensive database designed for integrating genomic and immunogenomic data related to tumors. The copy number variation (CNV) of CGB5 was analyzed across different malignancies using the TCGA dataset.

### 2.4 Survival analysis

The “survival” R package (3.3.1) was employed to perform proportional hazards survival regression analyses and hypothesis testing. Additionally, the “ggplot2” R package (version 3.3.6) and the “survminer” tool were utilized for result visualization. The prognostic significance of CGB5 in gastric cancer within the GEO dataset was analyzed using the Kaplan–Meier plotter (https://kmplot.com/analysis/) (14, 15). Detailed descriptions of additional data-preprocessing methodologies are provided in the Supplementary Materials Method.

### 2.5 Immune infiltration analysis

The TIMER database was employed to evaluate immune cell infiltration across 32 cancer types (https://cistrome.shinyapps.io/timer) (16, 17). The TISIDB database was utilized to investigate the association between CGB5 expression and immunological or molecular subtypes in various cancers. Furthermore, the SangerBox web tool (http://sangerbox.com/Tool) was utilized to analyze the expression of CGB5 and ESTIMATE scores and the presence of tumor-infiltrating immune cells across multiple tumor types.

### 2.6 Cell culture and treatment

The GES-1, SGC7901, and MNK45 lines were obtained from Cell Bank of the Experimental Center (Nanfang Hospital, Southern Medical University), and cultured in Dulbecco's Modified Eagle's Medium (C11995500BT, Thermo Scientific Gibco, USA). The medium was supplemented with 10% fetal bovine serum (catalog number 10099141, Thermo Scientific Gibco, USA). Cells were maintained in an incubator at 37 °C with 95% humidity and 5% CO_2_ (Thermo Fisher, China). Phosphate-buffered saline (C10010500BT, Thermo Scientific Gibco, USA) with a pH of 7.4 was used as the washing buffer in this study.

### 2.7 RNA extraction and real-time quantitative RT-PCR analysis

Total RNA was isolated from GES-1, MNK45, and SGC7901 cells using TRIzol reagent (15596018, Invitrogen, USA). The RNA concentration was measured using the NANO DROP 2000 (Thermo Fisher, USA). Reverse transcription was performed using the EvoM-MLV Mix Kit alongside gDNA Clean for qPCR (AG11728, Accurate Biology), and quantitative PCR amplification was carried out using the SYBR^®^ Green Premix Pro Taq HS qPCR Kit (Rox Plus; AG11718, Accurate Biology). The RT-qPCR process Adheres to the guidelines stipulated by the manufacturer. The results were normalized using the 2^−ΔΔ*Ct*^ technique. The dataset was further processed and analyzed by normalization against the internal GAPDH reference gene. The primer sequences used are listed in Table 1.

**Table 1:** Primers for the CGB5 and GAPDH genes.

**Genes**	**Sequences**
CGB5	F: GGCTACTGCCCCACCATG
	R: GAGCCACGGCGTAGGAGAC
GAPDH	F: ATCCCATCACCATCTTCCAGG
	R: GATGACCCTTTTGGCTCCC

### 2.8 Functional enrichment analysis

The present study utilized the Metascape database (http://metascape.org/gp/index.html#/main/step1) to perform functional enrichment analysis on the selected overlapping genes (18), which included Kyoto Encyclopedia of Genes and Genomes (KEGG) pathways, Gene Ontology (GO) terminology and tissue enrichment analysis. Genomic enrichment analysis (GSEA) was carried out using the clusterProfiler program (4.4.4) after converting molecular identifiers into suitable input data (19).

### 2.9 Statistical analysis

The Wilcoxon rank-sum test was employed to assess differences between the two groups, while the Spearman rank correlation test was utilized to evaluate relationships among the groups. Univariate and multivariate Cox proportional hazards regression analyses were performed to identify factors influencing prognosis. Kaplan–Meier analysis combined with the log-rank test was applied for survival analysis. Statistical analyses were performed using the R programming language (version 4.2.1), with statistical significance defined as *P*-values less than 0.05 (^*^P < 0.05, ^**^P < 0.01, ^***^P < 0.001, ^****^P < 0.0001).

## 3 Results

### 3.1 CGB5 pan-cancer expression

**Figure 1:**
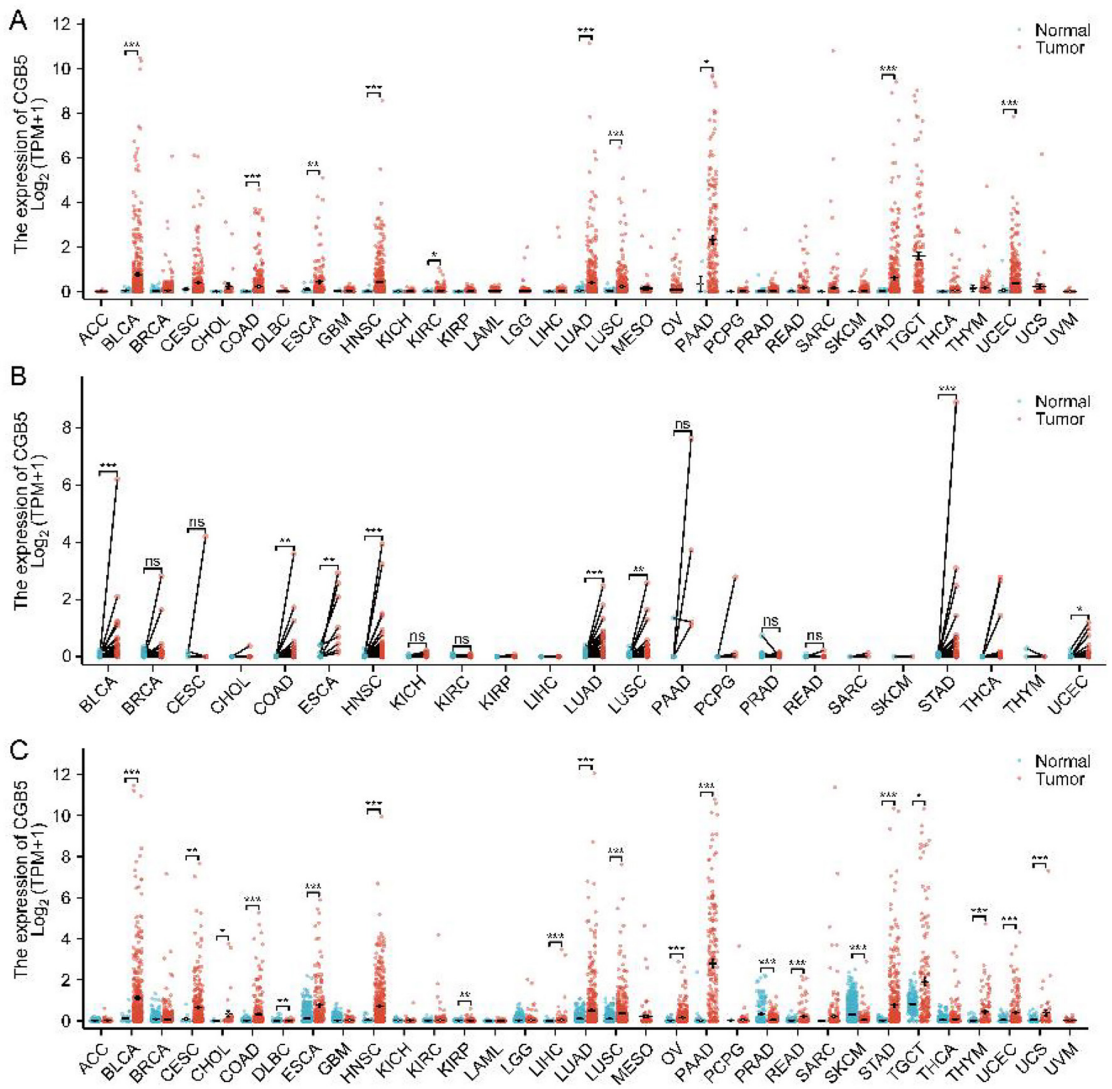
Illustrates CGB5 mRNA expression levels across various cancer types. **(A)** Illustrates the mRNA expression pattern of CGB5 across 33 distinct tumor types within the TCGA database. **(B)** CGB5 expression in matched samples of 23 tumors in the TCGA database. **(C)** Using TCGA_GTEx samples depicts the mRNA expression profile of CGB5 across 33 distinct tumors.

To investigate the mRNA expression levels of the CGB5 gene across various cancer types, we performed an analysis using TCGA harmonization data obtained from UCSC. The results demonstrated that CGB5 exhibited diverse expression patterns in most tumor types, with differential expression observed, including high expression, no significant differential expression, or low expression (Figure 1A). These findings were largely consistent with those derived from the TCGA_GTEx dataset (Figure 1C). Additionally, the expression of CGB5 was analyzed in 23 tumor types with paired samples in TCGA (Figure 1B).

**Figure 2:**
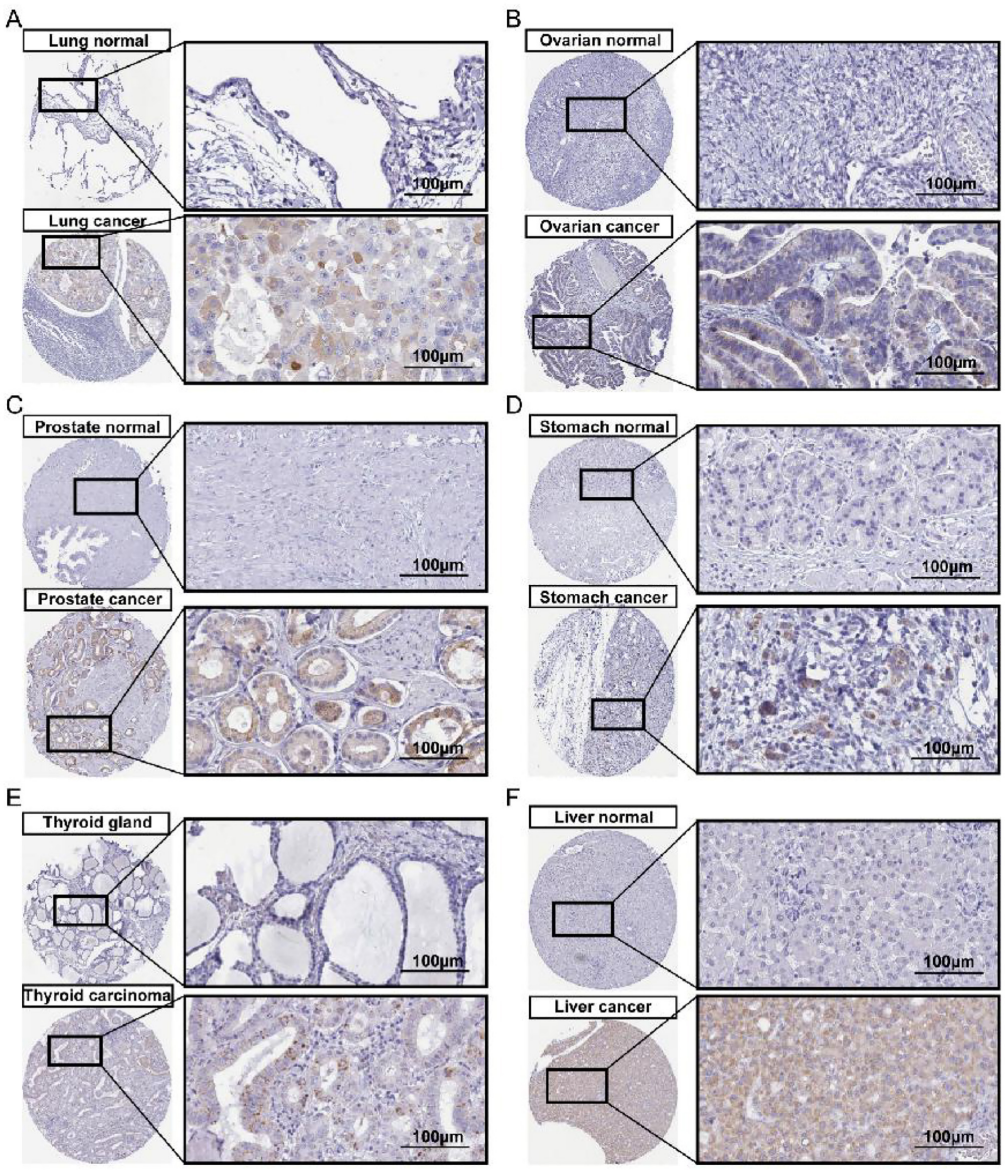
The protein expression level of CGB5 detected by IHC in pan-cancer extracted from HPA. **(A)** Lung cancer, **(B)** Ovarian cancer, **(C)** Prostate cancer, **(D)** Stomach carcinoma, **(E)** Thyroid carcinoma, **(F)** Liver cancer.

Furthermore, we evaluated the protein expression levels of CGB5 across multiple cancer types. Immunohistochemical analysis revealed that CGB5 exhibited significantly increased expression in six tumor tissues, including liver, lung, and gastric cancers, compared to normal control tissues (Figure 2). Additionally, using the UALCAN database, our results demonstrated that CGB5 expression was altered in BLCA, COAD, CESC, HNSC, LUSC, PAAD, STAD, UCEC, THCA, and THYM (Supplementary Figure S1).

Additionally, we evaluated CGB5 expression across sex subgroups. Significantly elevated CGB5 expression was observed exclusively in female UCEC patients (Supplementary Figure S2); however, as UCEC pathogenesis is restricted to females, this comparison lacks biological relevance. Altered expression of CGA (hCG-α subunit) was further detected in BRCA, ESCA, KICH, KIRC, KIRP, LUAD, LUSC, THCA, and UCEC (Supplementary Figure S3).

### 3.2 Mutations in CGB5 across different tumor types

**Figure 3:**
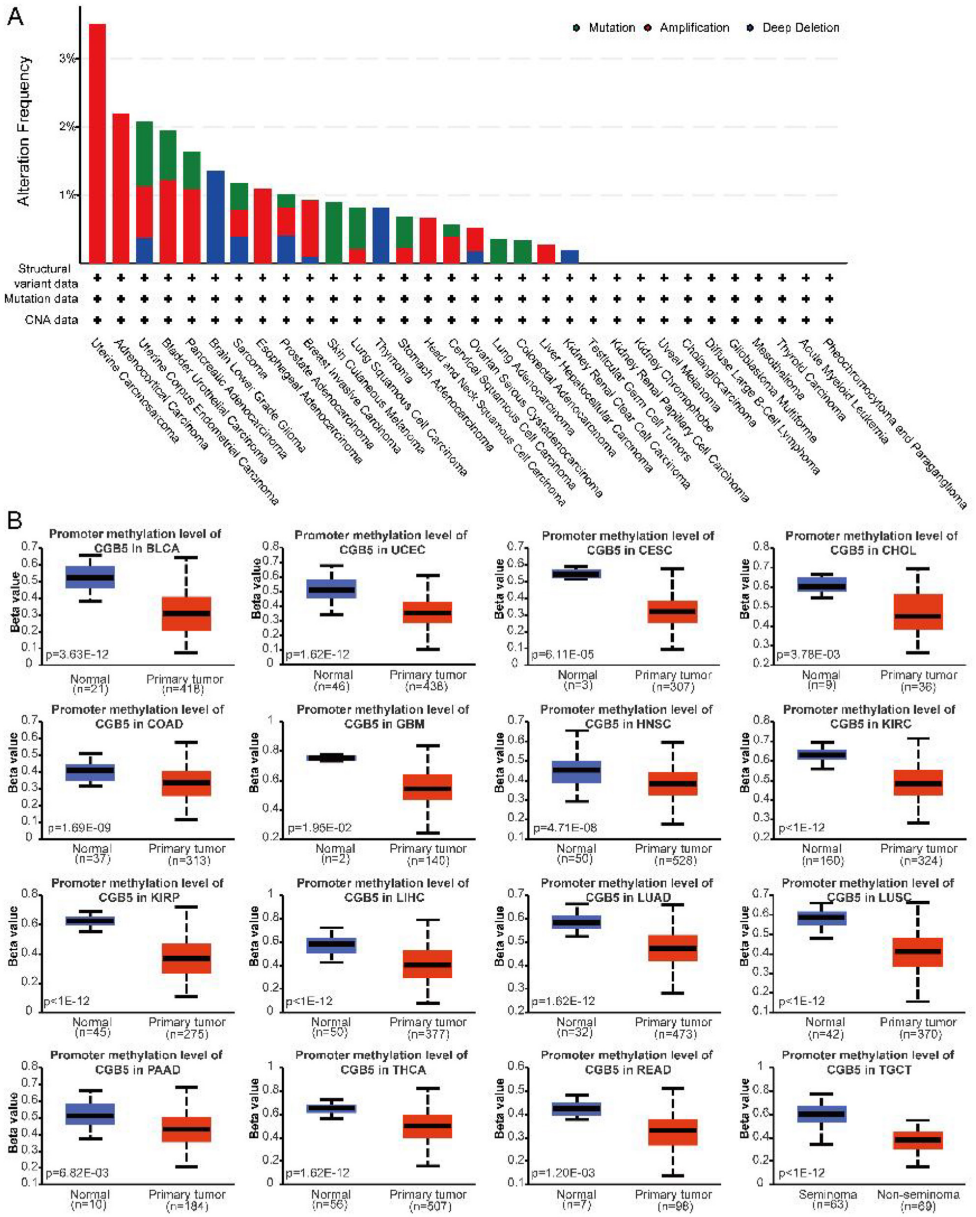
CGB5 gene mutations in diverse cancers. **(A)** Utilizing cBioPortal, the frequency of alterations across various mutation types in the CGB5 gene was visualized. **(B)** UALCAN tool was employed to analyze and compare the methylation levels of CGB5 gene in both normal and primary tumor tissues.

To investigate the mutation status of the CGB5 gene across various malignancies, its mutation profile was analyzed using the cBioPortal platform and TCGA data. Overall, the cancer analysis revealed that CGB5 exhibited a high amplification rate in UCS (>3%) and a significant mutation rate in UCEC (>1%). Brain Lower Grade Glioma demonstrated the highest frequency of “deep deletions” (>1%; Figure 3A). Furthermore, the promoter methylation levels of CGB5 were assessed using the TISIDB database. The results indicated notable reductions in CGB5 promoter methylation levels in specific tumor types, including BLCA, UCEC, CHOL, CESC, GBM, COAD, HNSC, KIRC, KIRP, LIHC, LUSC, LUAD, PAAD, THCA, READ, and TGCT (Figure 3B).

**Figure 4:**
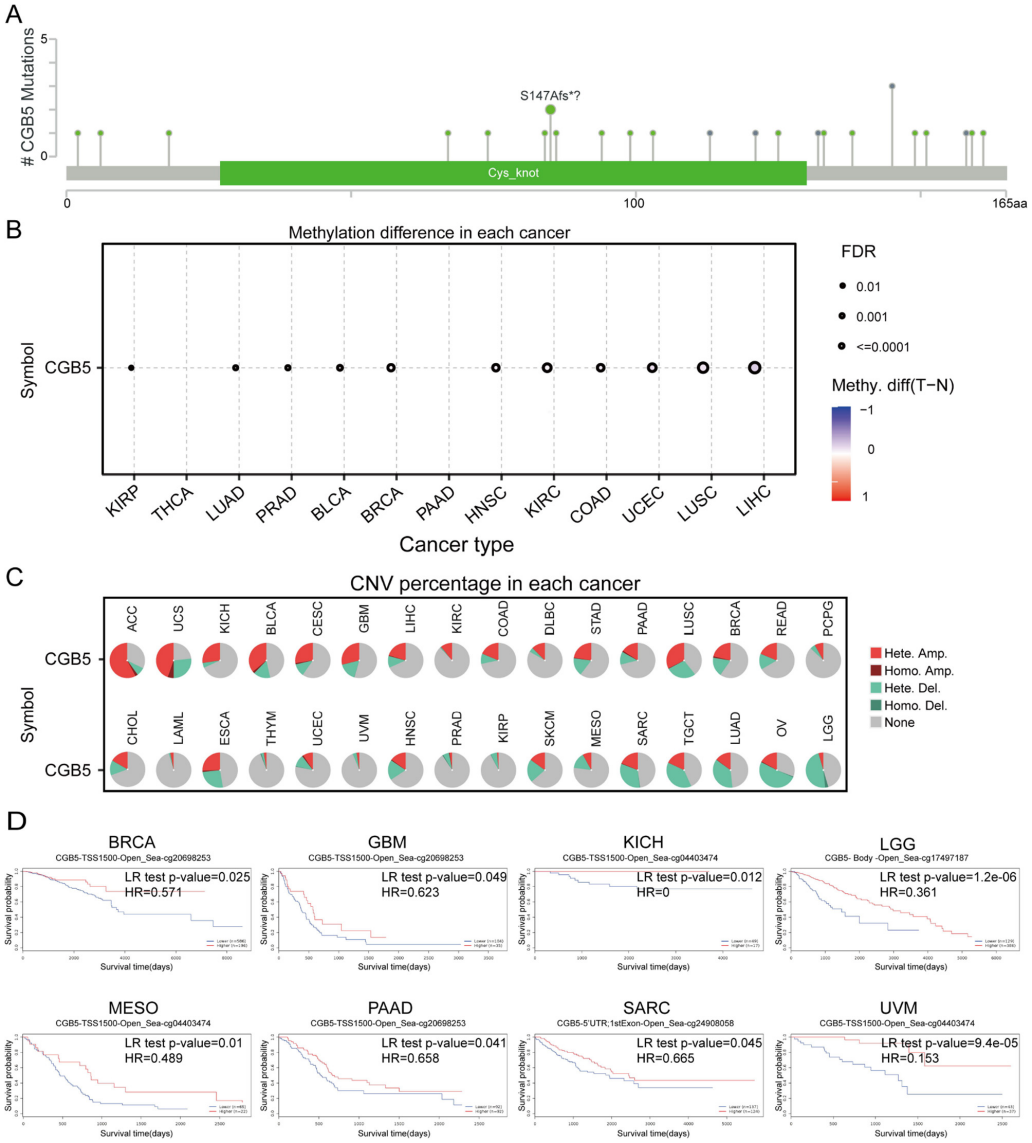
CGB5 gene mutation in pan-cancer. **(A)** Mutation site of CGB5. **(B)** Methylation difference of CGB5 in each cancer. **(C)** CGB5 CNV alterations in malignancies were evaluated using GSCA. **(D)** The OS difference between higher and lower methylation groups of CGB5 in the cancers.

Furthermore, we identified that the predominant types of mutations in CGB5 were missense and truncation mutations. For instance, the truncated S147Afs^*^? mutation could be detected (Figure 4A). Additionally, to evaluate the impact of DNA methylation, we performed a correlation analysis to investigate the relationship between CGB5 expression and DNA methylation within the promoter region (Figure 4B). We also assessed the contribution of copy number variations (CNVs) to CGB5 expression across different cancer types, with a higher proportion of CNVs observed in ACC and UCS (Figure 4C). To clarify the influence of CGB5 methylation on the prognosis of cancer patients, we conducted a prognostic analysis based on CGB5 methylation levels. The results indicated that reduced CGB5 methylation was associated with an unfavorable prognosis (Figure 4D).

### 3.3 The association between CGB5 expression and pan-cancer prognosis

**Figure 5:**
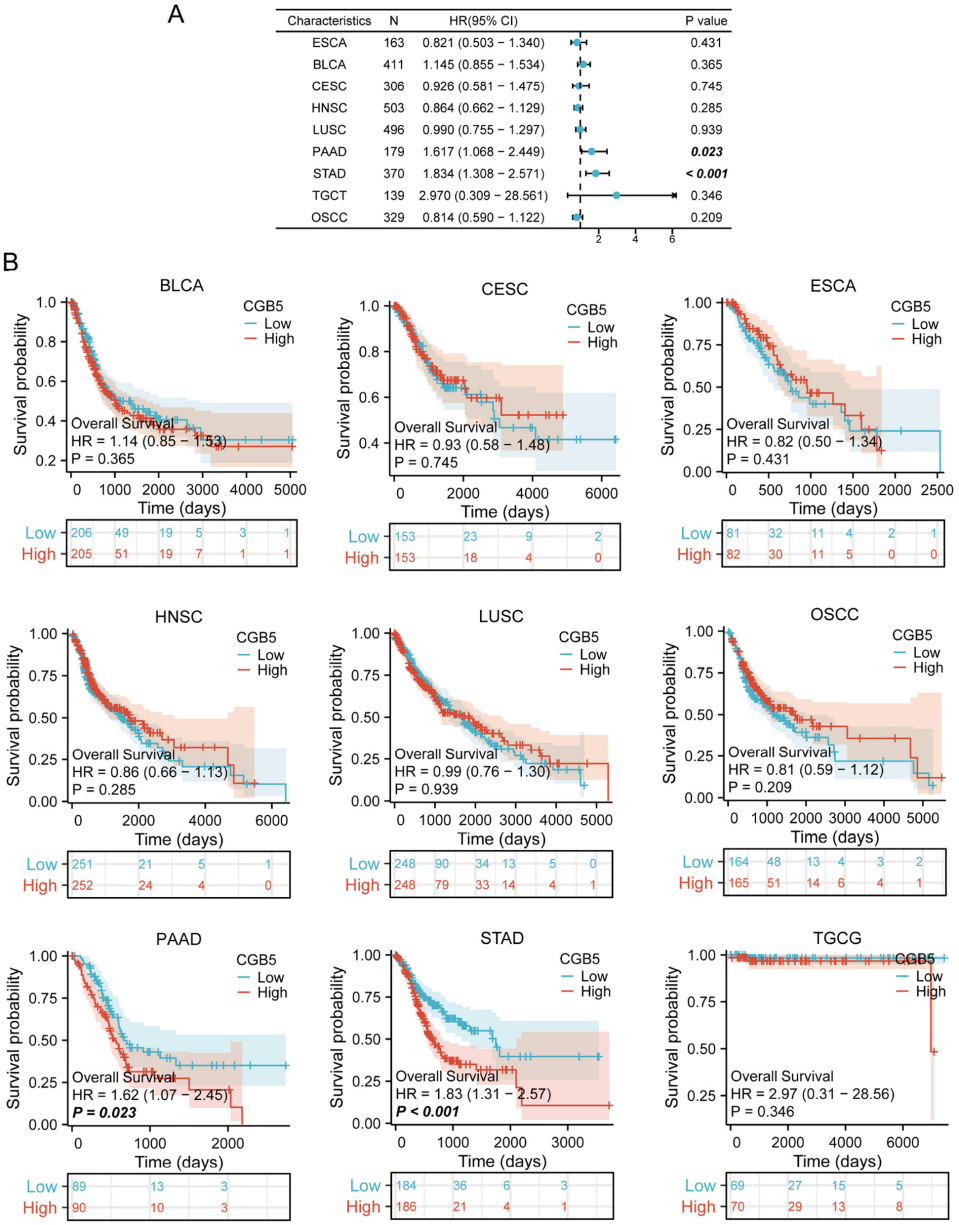
Correlation between CGB5 expression and OS in pan-cancer analysis. **(A)** The influence of CGB5 expression on OS across various malignancies is visually presented using a forest plot. **(B)** The effect of CGB5 expression on OS is separately illustrated for PAAD and STAD.

We conducted a Kaplan–Meier survival analysis to evaluate the prognostic significance of CGB5 across various cancer types. Additionally, this study investigated the correlation between CGB5 expression and clinical outcomes. Initially, we analyzed the association between CGB5 expression and OS in nine distinct cancer types (Figure 5A). The results demonstrated that abnormal CGB5 expression was significantly associated with OS in PAAD and STAD (Figure 5B), where increased CGB5 expression was linked to shorter OS in PAAD and STAD. Subsequently, we assessed the potential relationship between CGB5 expression and DSS (Supplementary Figure S4A). The findings revealed that CGB5 expression was correlated with DSS in PAAD and STAD (Supplementary Figure S4B), with high CGB5 expression being associated with poorer DSS in these cancers. Finally, we examined the relationship between CGB5 expression and PFI (Supplementary Figure S5A). The results indicated a significant association between CGB5 expression and PFI in PAAD and STAD (Supplementary Figure S5B), where elevated CGB5 expression was associated with poorer PFI in these cancer types. Furthermore, we conducted sex-stratified subgroup analyses for STAD and PAAD. In the TCGA cohort, CGB5 expression showed no significant association with OS in male STAD patients (Supplementary Figure S6A). However, elevated CGB5 expression correlated with poorer OS in the GEO cohort (Supplementary Figure S6G). Among female STAD patients, high CGB5 expression predicted worse prognosis in both TCGA and GEO datasets (Supplementary Figures S6D–F, J–L). Subgroup analysis of PAAD in TCGA similarly revealed that elevated CGB5 expression was associated with reduced OS in male patients (Supplementary Figure S7A). Notably, high CGB5 expression indicated poorer PFI regardless of sex (Supplementary Figure S7C, F). GEO data further demonstrated that increased CGB5 expression predicted worse OS and DFS in both male and female PAAD patients (Supplementary Figures S7G–J).

Concurrently, we evaluated the prognostic significance of CGA across cancers. Elevated CGA expression was associated with adverse outcomes in BLCA, KIRC, and UCEC (Supplementary Figure S8).

### 3.4 The correlation between CGB5 expression and the tumor immunological microenvironment

**Figure 6:**
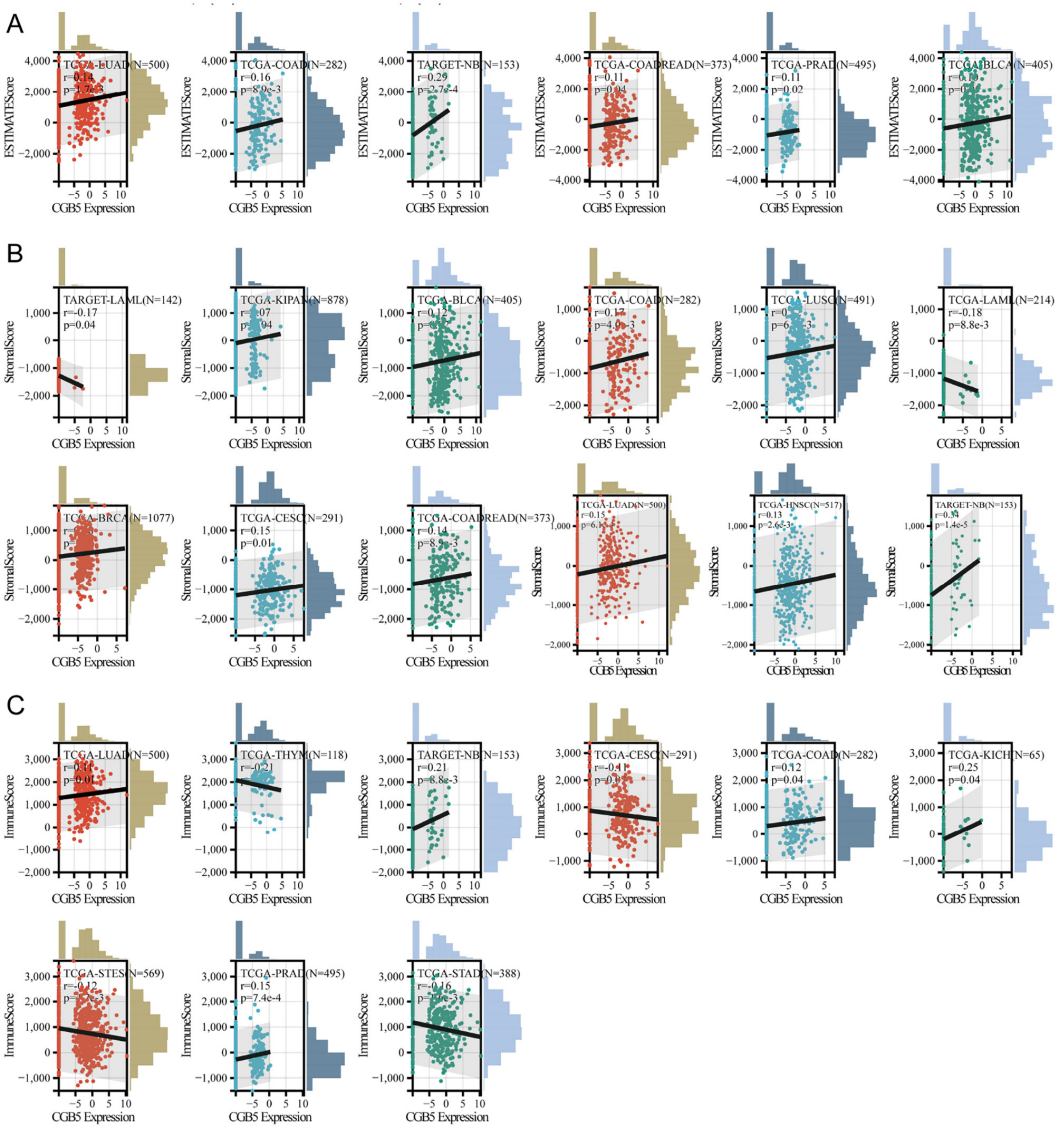
Immune infiltration of CGB5 in pan-cancer with estimate algorithm. **(A)** ESTIMATEScore, **(B)** StromalScore, **(C)** ImmuneScore.

The results demonstrated that CGB5 expression exhibits a significant association across various cancer types. We utilized diverse algorithms to investigate the potential correlation between CGB5 expression and the infiltration levels of various immune cell types across pan-cancer datasets. The correlations between B cells, cancer-associated fibroblasts (CAFs), CD8+ T cells, and CGB5 expression were comprehensively visualized in the heatmap (Supplementary Figure S9). Subsequently, we evaluated the TME characteristics of each sample using the ESTIMATE algorithm and compared the impact of CGB5 expression on TME-related metrics, including the ESTIMATEScore, ImmuneScore, and StromalScore, in pan-cancer cases with varying degrees of immune infiltration (Figure 6). From these findings, it is evident that CGB5 expression exhibited the strongest positive correlation with the stromal content and immune grade of tumor samples, while displaying a negative correlation with the immunological index.

**Figure 7:**
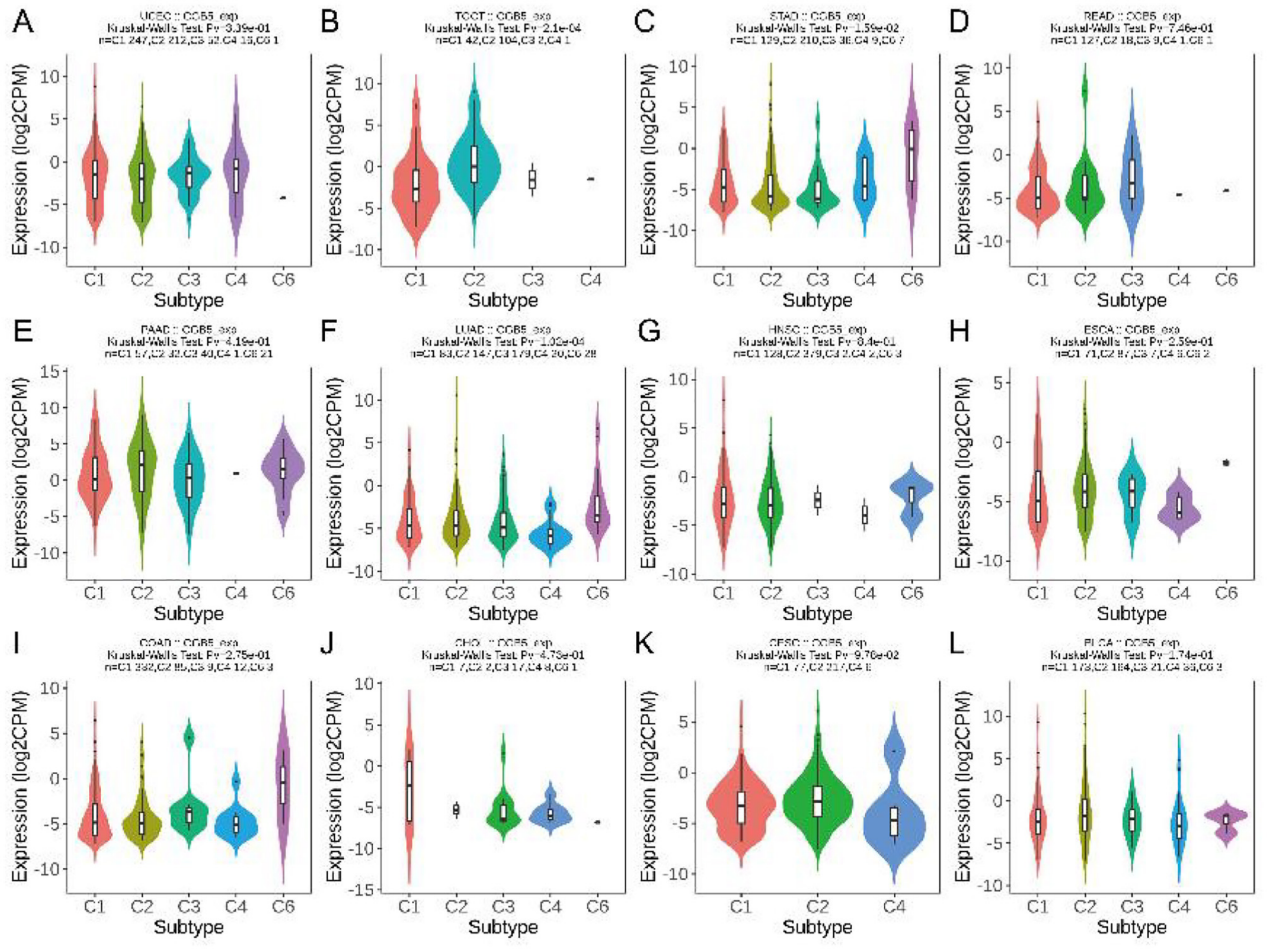
The association between CGB5 expression and pan-cancer immune subtypes. **(A)** UCEC, **(B)** TGCT, **(C)** STAD, **(D)** READ, **(E)** PAAD, **(F)** LUAD, **(G)** HNSC, **(H)** ESCA, **(I)** COAD, **(J)** CHOL, **(K)** CESC, **(L)** BLCA

Furthermore, we assessed the correlation between CGB5 expression and immune cell infiltration across pan-cancer datasets using multiple algorithms, including TIMER, EPIC, QUANTISEQ, XCELL, CIBERSORT, MCPCOUNTER, IPS, and Quantiseq (Supplementary Figures S10–S12). As demonstrated in the results, CGB5 infiltration levels varied significantly across different malignant conditions depending on the algorithm used. This observation underscores the intricate relationship between CGB5 and the TME. Subsequently, we conducted an analysis to evaluate the influence of CGB5 expression on immunological and molecular subgroups across pan-cancer types. The immune subgroups were categorized into six distinct clusters: C1 (wound-healing), C2 (IFN-gamma-dominant), C3 (inflammatory), C4 (lymphocyte-depleted), C5 (immunosuppressed), and C6 (TGF-beta-dominant). Despite quantitative variations across algorithms, we observed a consistent cross-method trend: elevated CGB5 expression positively correlated with tumor-progressive immune microenvironment phenotypes, including increased stromal content (ESTIMATE StromalScore) and immunosuppressive subtypes (e.g., C4 lymphocyte-depleted subtype) (Figures 6, 7).

### 3.5 High expression levels of CGB5 associated with a poor prognosis in GC patients

**Figure 8:**
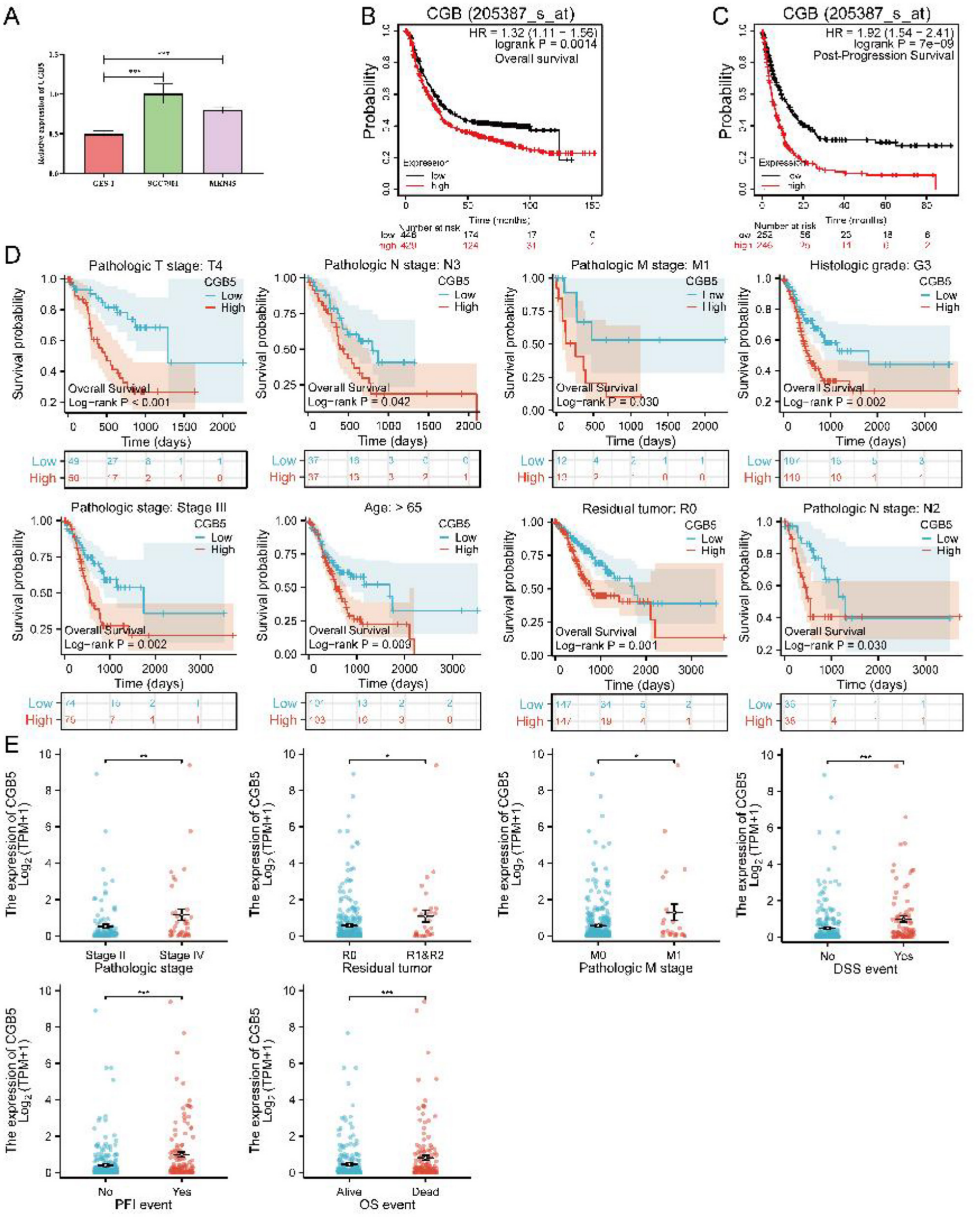
The key role of CGB5 in GC. **(A)** The expression of CGB5 is significantly higher in GC cell lines SGC7901 and MNK45 compared to the gastric mucosal epithelial cell line GES-1. **(B, C)** High CGB5 expression is associated with poor prognosis in GC patients, as evidenced by a compiled dataset (GSE14210, GSE15459, GSE62254, GSE22377, GSE29272, and GSE51105) from the GEO database. **(D)** Subgroup survival analyses of CGB5 in GC. **(E)** The correlation between CGB5 expression and clinical features in GC.

The results of pan-cancer analysis indicate that CGB5 exhibits significant prognostic value in GC. Consequently, we conducted a comprehensive investigation into the role of CGB5 within the context of GC. First, we confirmed the up-regulation of CGB5 expression in gastric cancer cells through experimental validation (Figure 8A). Additionally, the association between high CGB5 expression and poor prognosis in GC was validated using the GEO database (Figures 8B, C). Subsequent subgroup analyses revealed a notable correlation between elevated CGB5 expression and unfavorable OS outcomes in the following subgroups of GC patients: age > 65 years (*P* = 0.009), histological grade 3, pathological stage III, pathological T4 stage, pathological N2 stage, pathological M1 stage, and absence of residual tumor (Figure 8D). Furthermore, increased CGB5 expression was significantly associated with DSS and PFI in GC patients, particularly in relation to clinical characteristics such as age, histological grading, pathological staging, and disease progression (Supplementary Figure S13). Moreover, CGB5 expression was demonstrated to be strongly correlated with the clinical features of GC patients. Specifically, in the context of advanced disease stages, high CGB5 expression may serve as a predictor of disease progression and poor prognosis (Figure 8E).

### 3.6 Functional enrichment analysis of CGB5 in GC

**Figure 9:**
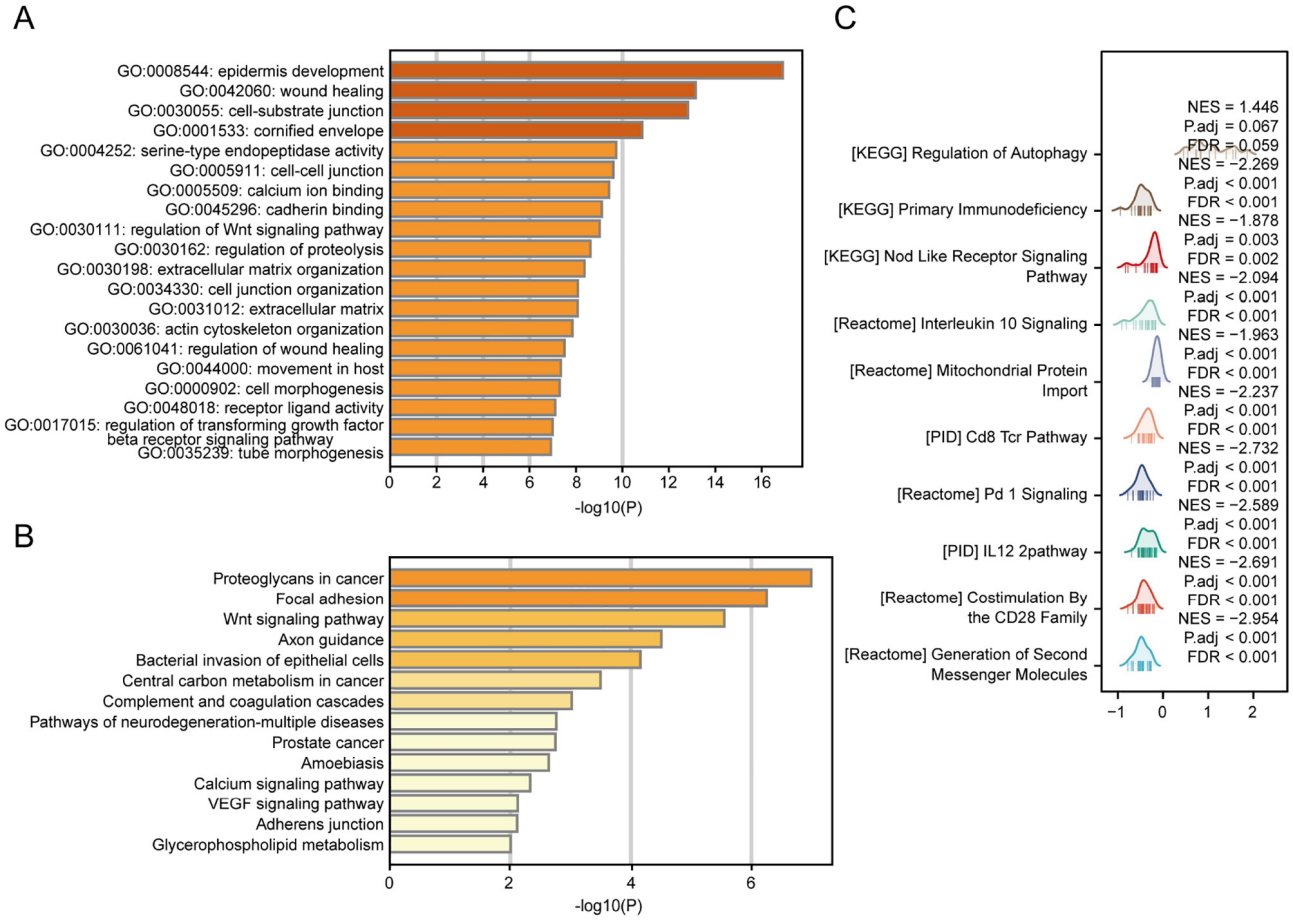
Functional enrichment analysis of co-expressed CGB5 genes in GC. **(A)** GO enrichment analysis of genes co-expressed with CGB5 in GC. **(B)** KEGG enrichment analysis of genes co-expressed with CGB5 in GC. **(C)** GSEA of CGB5 co-expressed genes in GC reveals enriched signaling pathways.

To elucidate the biological function of CGB5 in tumors, we conducted a further analysis of the 100 genes most highly correlated with CGB5. GO analysis revealed that genes associated with CGB5 may be involved in molecular functions related to “epidermis development,” “cell-substrate junction,” “cornified envelope,” and “regulation of the Wnt signaling pathway” (Figure 9A). KEGG pathway analysis indicated that CGB5-related genes may play roles in pathways such as “Proteoglycans in cancer,” “Focal adhesion,” “Wnt signaling pathway,” and “Central carbon metabolism in cancer” (Figure 9B). Furthermore, to investigate the biological function of CGB5 in GC, we performed GSEA based on differential expression analysis of CGB5 to gain deeper insights into its role. The results demonstrate that CGB5 is predominantly associated with immune-related pathways, including “Primary immunodeficiency,” “CD8 TCR signaling pathway,” and “PD-1 checkpoint signaling” (Figure 9C).

## 4 Discussion

Our pan-cancer analysis demonstrates that aberrant CGB5 expression significantly correlates with poor prognosis across multiple cancer types, specifically in malignancies like GC and PAAD, potentially driven by genetic mutations, DNA methylation, and CNAs. Notably, CGB5 exhibits strong associations with immune cell infiltration, with distinct patterns and magnitudes observed in specific cancers. In GC, we validated CGB5 upregulation and further established its independent adverse impact on patient survival. Mechanistically, CGB5 over-expression likely influences tumor progression not only through modulation of immune infiltration but also via interactions with multiple signaling pathways. Collectively, this multi-dimensional study underscores the potential oncogenic role of CGB5 and provides a rationale for targeting CGB5 in future therapeutic strategies.

This investigation concurrently evaluated CGA (hCGα-encoded protein) to establish CG5′s functional specificity. Tumor-specific dysregulation of CGA significantly influenced prognosis across malignancies, with minimal overlap in affected cancer types between CGA and CGB5. Sex-stratified analyses further confirmed sex-independent associations between CGB5 overexpression and adverse outcomes, highlighting its tumor-specific functional profile and clinical translational potential.

Gene expression levels are influenced by numerous factors, among which genetic variations and epigenetic modifications are included (20, 21). It is notable that environmental exposures have the potential to induce epigenetic alterations, which can impact gene expression and modify the risks of diseases related to genetic variations (22). Studies suggest that CIAPIN1 overexpression promotes breast cancer by regulating cell cycle and DNA replication, while DBNDD1 hypomethylation may contribute to tumor progression by affecting DNA synthesis or damage repair (23, 24). In this study, we investigated the gene mutations and CNVs of the CGB5 gene across pan-cancer types. Gene mutations result in alterations in DNA sequences, thereby influencing the structure and function of encoded proteins. Such changes may give rise to abnormal protein functions, thereby disturbing cellular processes and gene expression regulation (25, 26). CNVs refer to the deletion or duplication of DNA segments, leading to an imbalance in gene dosage (27, 28). When genes are duplicated, the corresponding mRNA and protein levels may increase, resulting in overexpression. Conversely, gene deletions may cause a reduction in mRNA and protein production, leading to underexpression (29). Conclusively, both gene mutations and CNVs can significantly affect gene expression by influencing transcriptional regulation, altering the binding of transcription factors, and affecting mRNA stability, ultimately leading to changes in the abundance of specific gene transcripts (30–32). In addition, the methylation status of the CGB5 promoter exhibits variations among different tumor types. DNA methylation is a crucial epigenetic modification that primarily occurs at CpG dinucleotides, especially within CpG islands located in the promoter regions of genes (33, 34). High methylation levels in CpG islands can inhibit the interaction between transcription factors and DNA, thereby repressing gene transcription (35). In our study, this is supported by the negative correlation between CGB5 methylation and mRNA expression (Figure 4B), which may promote oncogenic signaling pathways, such as Wnt signaling, or immune suppression via PD-1 pathway activation (Figure 9C). These findings suggest that CGB5 methylation status could serve as a prognostic biomarker, with hypomethylation indicating aggressive tumor behavior. This mechanism provides important clues for understanding the changes in gene expression regulation during tumorigenesis. Through comprehensive analysis of gene mutations, CNVs, and DNA methylation status, we can more comprehensively reveal the role of the CGB5 gene in cancer and its potential biological significance (36).

An expanding body of research has underscored the critical role of the immune microenvironment in tumor initiation and progression. Studies have shown that the pan-immune inflammation value and albumin-to-globulin ratio are closely associated with the tumor immune microenvironment and serve as important prognostic indicators in colorectal cancer (37, 38). In our study, we observed a robust correlation between CGB5 expression across various cancer types and immune cell infiltration. The results derived from the estimation algorithm demonstrated that CGB5 expression was positively correlated with the tumor estimate score and stromal score but not significantly associated with the immune score. Typically, higher immune scores indicate a greater abundance of immune cells, which may contribute to an effective antitumor immune response (39). However, in this study, high CGB5 expression exhibited an inverse correlation with immune cell infiltration in different tumor types. Moreover, elevated EstimateScores and StromalScores in tumors generally reflect a higher proportion of stromal and non-immune cells within the TME, which may promote tumor progression without improving patient outcomes (40).

Compared with T cells, CGB5 expression exhibited a stronger positive correlation with the infiltration of B cells and cancer-associated fibroblasts (CAFs). Although direct mechanistic studies delineating the interaction between CGB5 and B cells remain scarce, accumulating evidence indicates that hCG family members can exert immunomodulatory effects on B cells. In a previous study, mucosal administration of Lactobacillus engineered to express hCGβ elicited robust anti-hCGβ antibody responses, indicating that trophoblastic antigens can effectively prime and activate B cells (41). This observation implies that aberrant CGB5 expression within the tumor microenvironment may similarly trigger B cell mediated immune responses. CAFs constitute a dominant stromal component of the tumor microenvironment, exerting pivotal roles through ECM remodeling, cytokine signaling modulation, and regulation of immune cell functionality. Accumulating evidence indicates that CGB5 and other hCG β-subunits can promote the conversion of resident fibroblasts into CAFs. Specifically, CGB5 disrupts canonical TGF-β signaling, thereby driving fibroblasts to acquire a CAF phenotype characterized by robust secretion of ECM proteins and pro-inflammatory cytokines (42), ultimately remodeling the ECM and facilitating tumor invasion (41, 43). Concurrently, these secreted factors recruit and activate stromal cells- including CAFs -to establish an inflammatory and fibrotic niche that further promotes immune evasion (42, 44).

For tumors to survive and progress, they must circumvent immune surveillance. CGB5-expressing tumor cells convert conventional CD4^+^ T cells into Tregs by up-regulating the immunosuppressive cytokines TGF-β and IL-10, together with the transcription factor FOXP3. These Tregs, via cell-to-cell contact and the release of IL-10 and TGF-β, suppress the functions of effector T cells and other immune subsets, thereby attenuating antitumor immunity. Mechanistic studies further demonstrate that exposing tumor cells to hCG augments FOXP3 expression and increases Treg differentiation, indicating that CGB5-mediated signaling is a key driver of the immune-evasion phenotype (41).

In recent years, immune-checkpoint inhibitors (ICIs) targeting molecules such as PD-L1 and CTLA-4 have substantially reshaped the therapeutic landscape for a broad spectrum of malignancies. However, the efficacy of these agents is critically contingent upon a pre-existing and robust antitumor immune response. For example, in the phase III IMvigor210 trial evaluating atezolizumab for urothelial carcinoma, patients with high CGB-family gene expression exhibited markedly reduced CD8^+^ T-cell infiltration and significantly shorter overall survival, indicating that these tumors are intrinsically less responsive to immune-checkpoint inhibition (42, 45). Collectively, these molecular events orchestrate an immune-evasive phenotype that enables tumor cells to sustain proliferation despite ongoing host immune surveillance.

Encouragingly, pre-clinical evidence now substantiates the clinical potential of CGB5-directed interventions. Vaccination protocols or antibody-based neutralization of hCGβ markedly attenuated tumor growth in experimental models, indicating that disruption of CGB5-dependent pathways can re-invigorate antitumor immunity and enhance responsiveness to immune-checkpoint blockade (41, 46). From a clinical standpoint, CGB5 exerts dual functions—driving intrinsic tumor aggressiveness while profoundly reshaping the immune microenvironment–thereby serving as both a prognostic biomarker and a compelling therapeutic target. Patients with elevated CGB5 expression can be stratified into a high-risk subset anticipated to derive limited benefit from monotherapy with immune checkpoint inhibitors, thus warranting investigation of rational combination regimens: first, combinatorial targeting of CGB5 or its downstream effectors—exemplified by TGF-β inhibitors and anti-IL-10 antibodies—together with immune-checkpoint inhibitors is anticipated to dismantle the immunosuppressive fortress. Second, circulating HCG (including CGB5-derived species) is readily quantifiable in plasma or urine, offering a non-invasive liquid-biopsy companion for real-time monitoring of therapeutic response and disease progression.

Previous studies have explored the role of CGB5 in gastric cancer, primarily focusing on gene set analyses while offering limited insight into its underlying mechanisms (7, 47, 48). In this study, we confirmed the robust expression of CGB5 in gastric cancer cells and further investigated its mechanism of action. Our results demonstrated that CGB5 facilitates gastric cancer progression via multiple pathways, with significant enrichment observed in the Wnt signaling pathway through both GO and KEGG analyses. The Wnt signaling pathway plays a critical role in various physiological processes, and its dysregulation is commonly implicated in gastric cancer. Aberrant activation of this pathway contributes to chemotherapy resistance, thereby promoting gastric carcinogenesis and resistance development, which highlights its significance in gastric cancer progression (49). Recent studies show that the Wnt signaling pathway reduces lipid peroxidation by up-regulating GPX4, an essential antioxidant enzyme that prevents cellular iron oxidation, ultimately inhibiting ferroptosis in gastric cancer cells (50). Furthermore, GSEA analysis suggests that CGB5 may promote gastric cancer progression by modulating the immune microenvironment, underscoring the pivotal role of immune infiltration in gastric cancer, findings that are consistent with prior research (8, 47).

This study possesses certain limitations that merit acknowledgment. First, the findings are primarily based on publicly accessible data from online databases rather than direct clinical observations, which may raise questions about the reliability of the data. To address this concern, we prioritized the use of large-scale and well-established databases, such as TCGA and GEO. Second, although CGB5 expression was confirmed in GC cell lines in this study, functional validation and further investigations in additional tumor types(such as PAAD) demand comprehensive *in vitro* and *in vivo* experiments utilizing relevant cancer cell lines and/or animal models. Moreover, discrepancies in algorithms or data sources across different databases could potentially lead to inconsistencies in the results. The inherent complexity of the TME necessitates acknowledgment of methodological constraints in immune cell quantification. Differences in computational frameworks—such as CIBERSORT's deconvolution-based model vs. XCELL's reliance on cell type-specific gene signatures—may inherently yield inter-algorithm variability for identical immune metrics. Concurrently, the bulk RNA-seq approach utilized here lacks spatial resolution to resolve CGB5 expression dynamics within specific cellular subpopulations or characterize cell-cell interactions. Consequently, we advocate future validation through scRNA-seq technology in tractable malignancies demonstrating significant CGB5 associations (e.g., GC or PAAD). This would specifically interrogate CG5′s functional modulation within cancer-associated fibroblasts and T-cell subpopulations, thereby advancing mechanistic insights for developing precision immunotherapies targeting CGB5. In summary, future studies should aim to overcome these limitations by conducting large-scale clinical trials or employing multi-source data integration for thorough validation.

## 5 Conclusion

To summarize, CGB5 expression was significantly up-regulated across a range of malignancies and exhibited a robust association with the prognoses of cancer patients, specifically in malignancies like GC and PAAD. This observation not only highlights its essential role in cancer development but also demonstrates a close association with the prognosis of cancer patients, specifically in malignancies like GC and PAAD. Extensive studies indicate that CGB5 expression in tumor tissues may facilitate tumorigenesis and progression by modulating the tumor immune microenvironment. Moreover, our experimental findings have further confirmed the elevated expression of CGB5 in GC and clarified its correlation with immune cell infiltration. Specifically, the expression level of CGB5 appears to directly affect the distribution and functional state of immune cells within the tumor microenvironment, offering valuable clues for elucidating the mechanisms underlying tumor immune evasion. Clinically, CGB5 expression could be integrated into diagnostic panels to stratify patients at higher risk of poor outcomes. Moreover, to validate CGB5′s role in immunotherapy response, future experiments could employ CRISPR/Cas9-mediated CGB5 knockout in cancer cell lines to evaluate changes in immune cell infiltration and response to ICIs.

## Data Availability

The original contributions presented in the study are included in the article/Supplementary material, further inquiries can be directed to the corresponding authors.
