# Supplementary material for: Comprehensive pan-cancer analysis reveals CGB5 is a potential promising predictive and immunotherapeutic biomarker
Source: Front Med (Lausanne). 2025 Sep 19;12:1624815. doi: 10.3389/fmed.2025.1624815 (PMC12491329; doi:10.3389/fmed.2025.1624815)

**Figure S1. The protein expression level of CGB5 in pan-cancer.** The results showing CGB5 overexpression in (A). BLCA, (B). CESC, (C). COAD, (D). HNSC, (E). LUSC, (F). PAAD, (G). STAD, (H). UCEC, (I). THCA, (J). THYM.


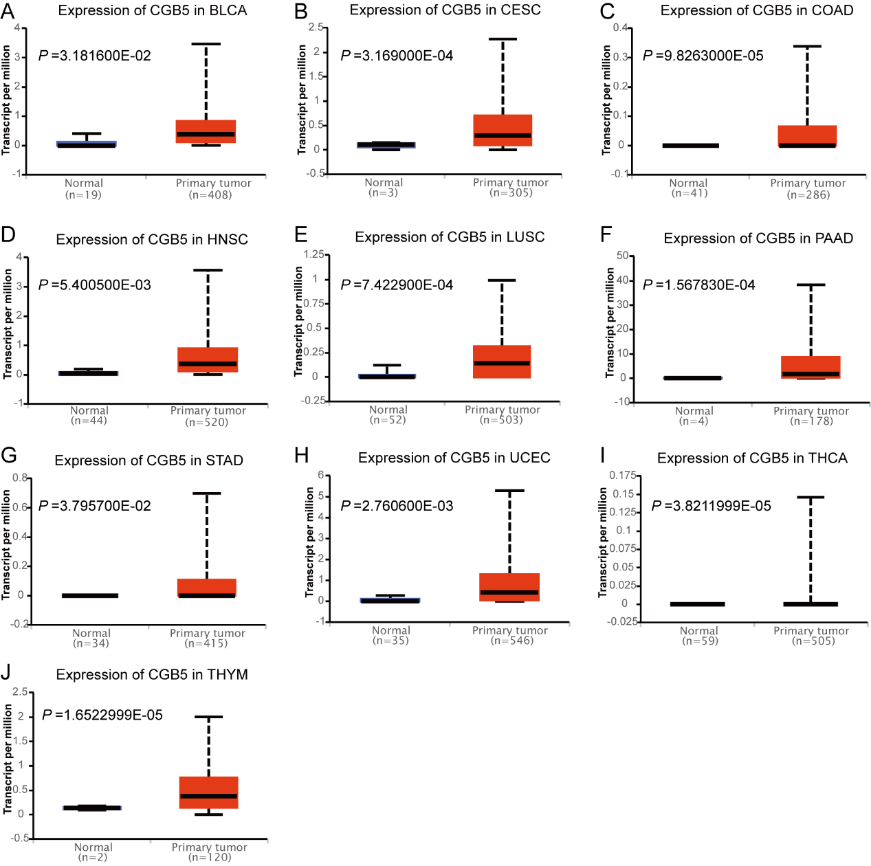


**Figure S2.Subgroup analysis of CGB5 expression by gender in pan-cancer.**

**
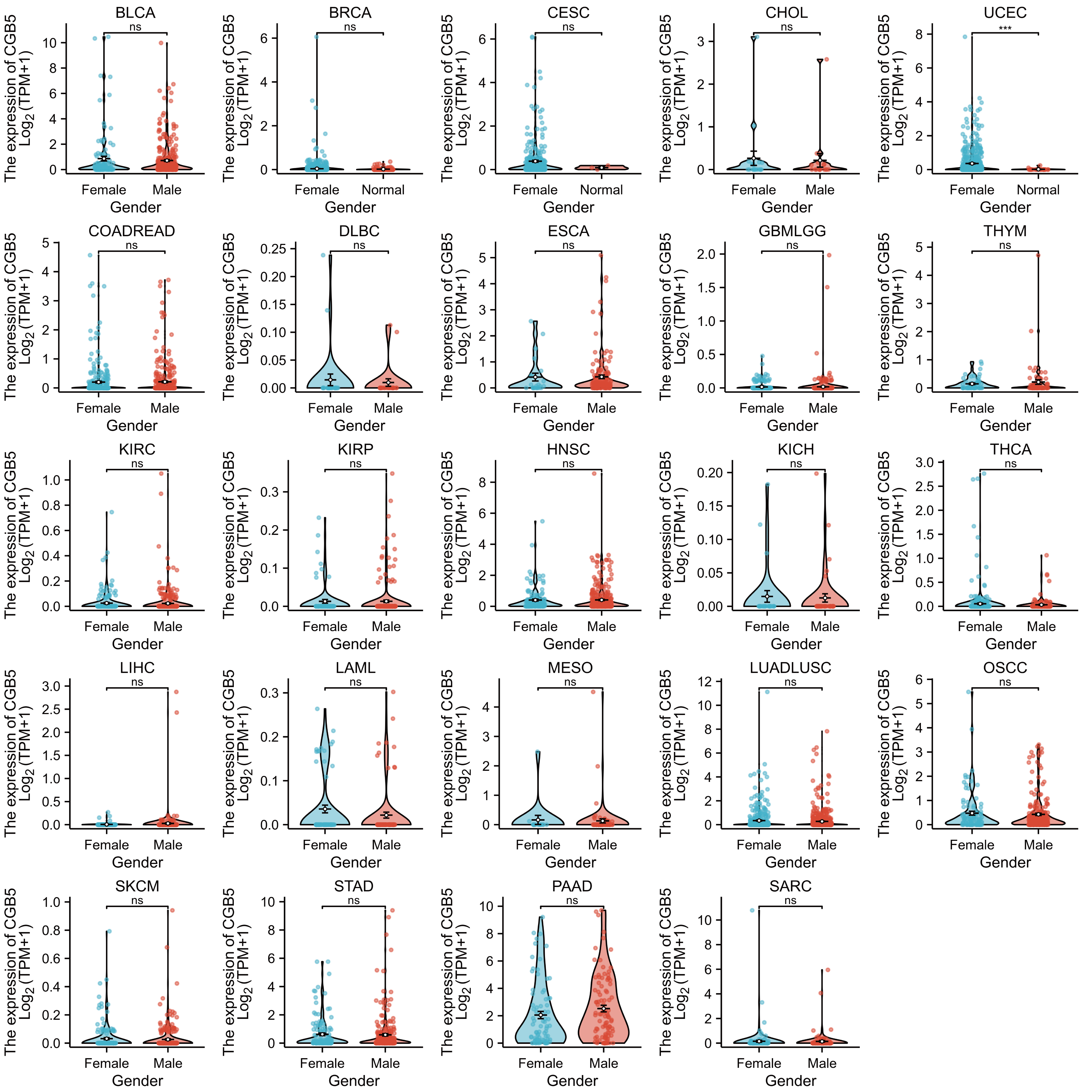
**

**Figure S3. The mRNA expression level of CGA in pan-cancer.**

**
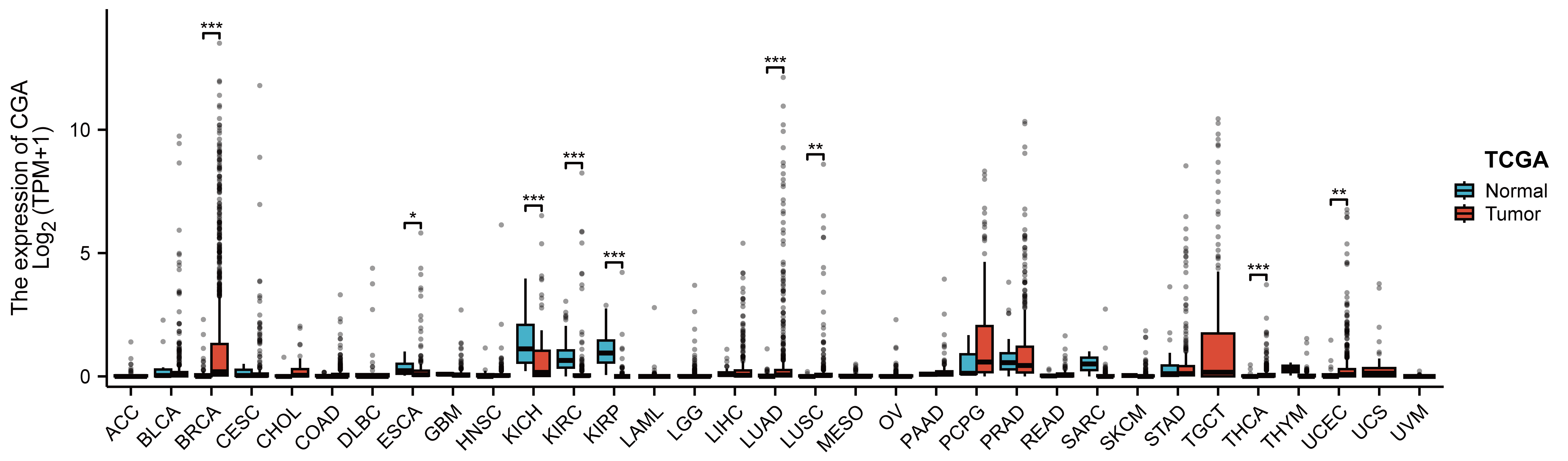
**

**Figure S4**. **Prognostic Significance of CGB5 Expression on DSS in Pan-Cancer Analysis.** (A) Multivariate Cox regression forest plot demonstrating HR for CGB5-high vs. CGB5-low groups across 9 cancer types (TCGA cohort). (B) Survival curves separately demonstrate the impact of CGB5 expression on DSS in pan-cancer.


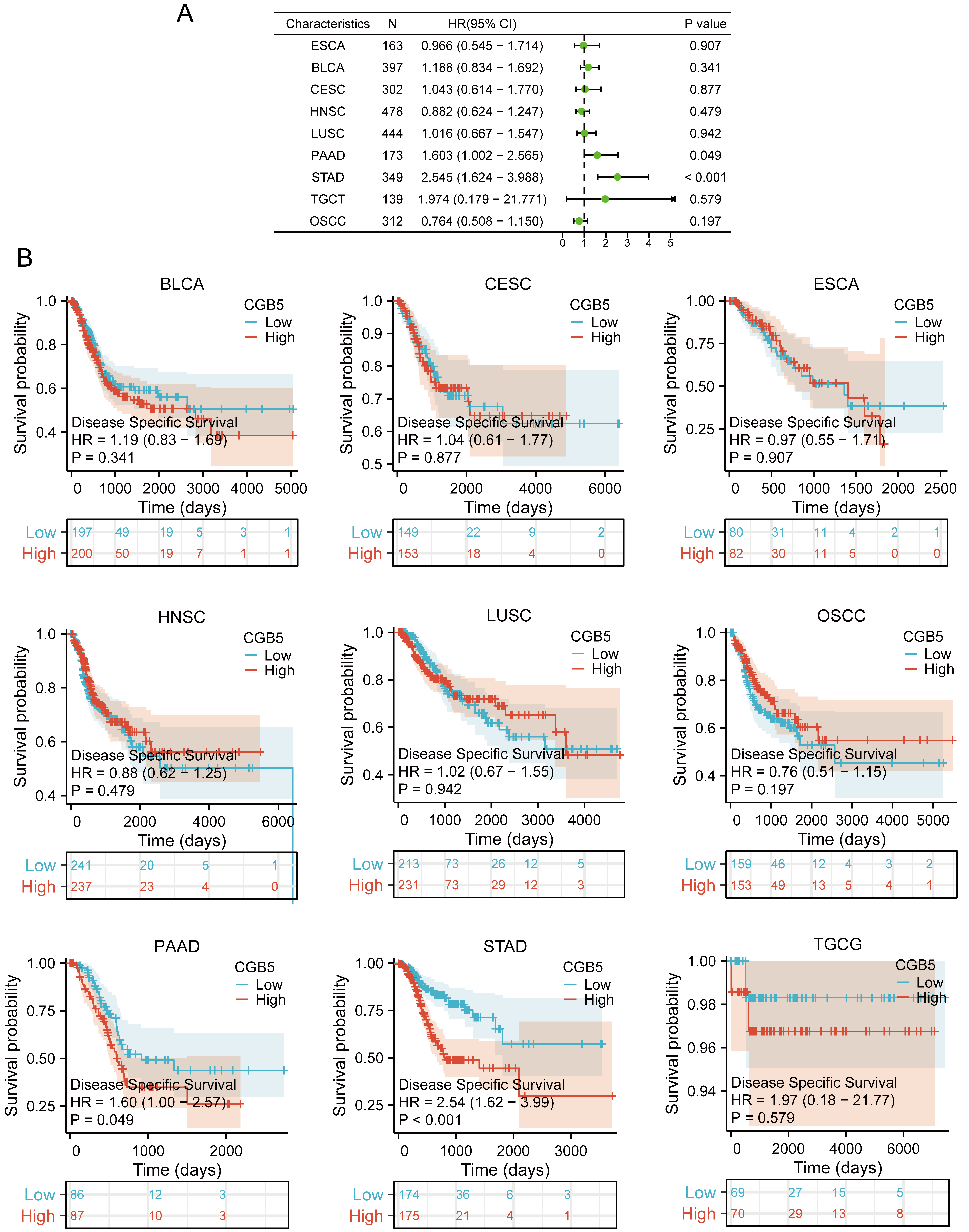


**Figure S5. Influence of CGB5 Expression on PFI in Pan-Cancer Analysis.** (A) The impact of CGB5 expression on PFI within the TCGA dataset is illustrated using a forest plot. (B) The effect of CGB5 expression on PFI is separately demonstrated for pan-cancer.


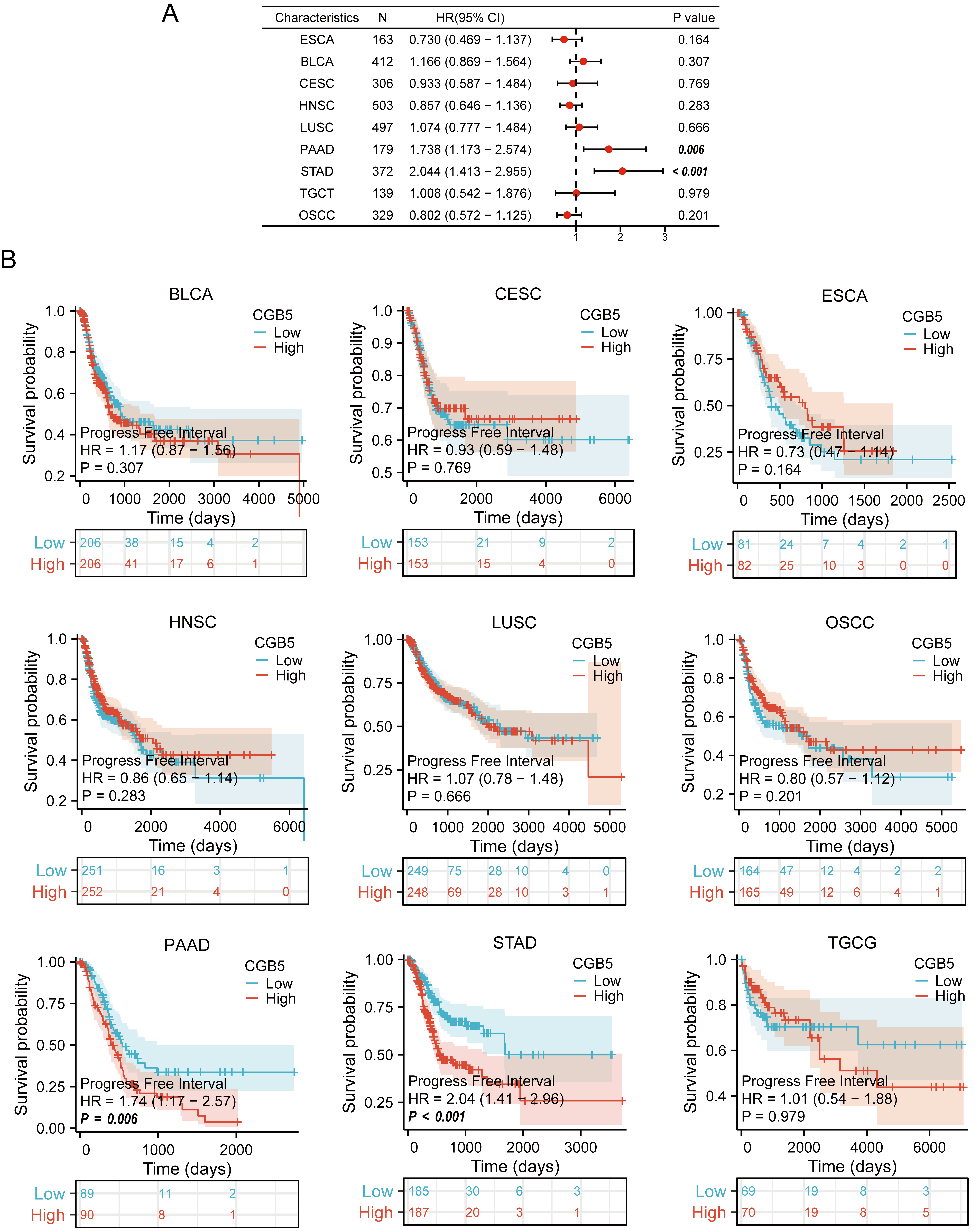


**Figure S6. Subgroup analysis of CGB5 in STAD prognosis by gender.(A-C)**The impact of CGB5 expression on OS,PFI,DSS of male STAD patients in TCGA database.**(D-F)**The impact of CGB5 expression on OS,PFI,DSS of female STAD patients in TCGA database.**(G-I)**The impact of CGB5 expression on OS,FP,PPS of male STAD patients in GEO database.**(J-L)**The impact of CGB5 expression on OS,FP,PPS of female STAD patients in GEO database.

**
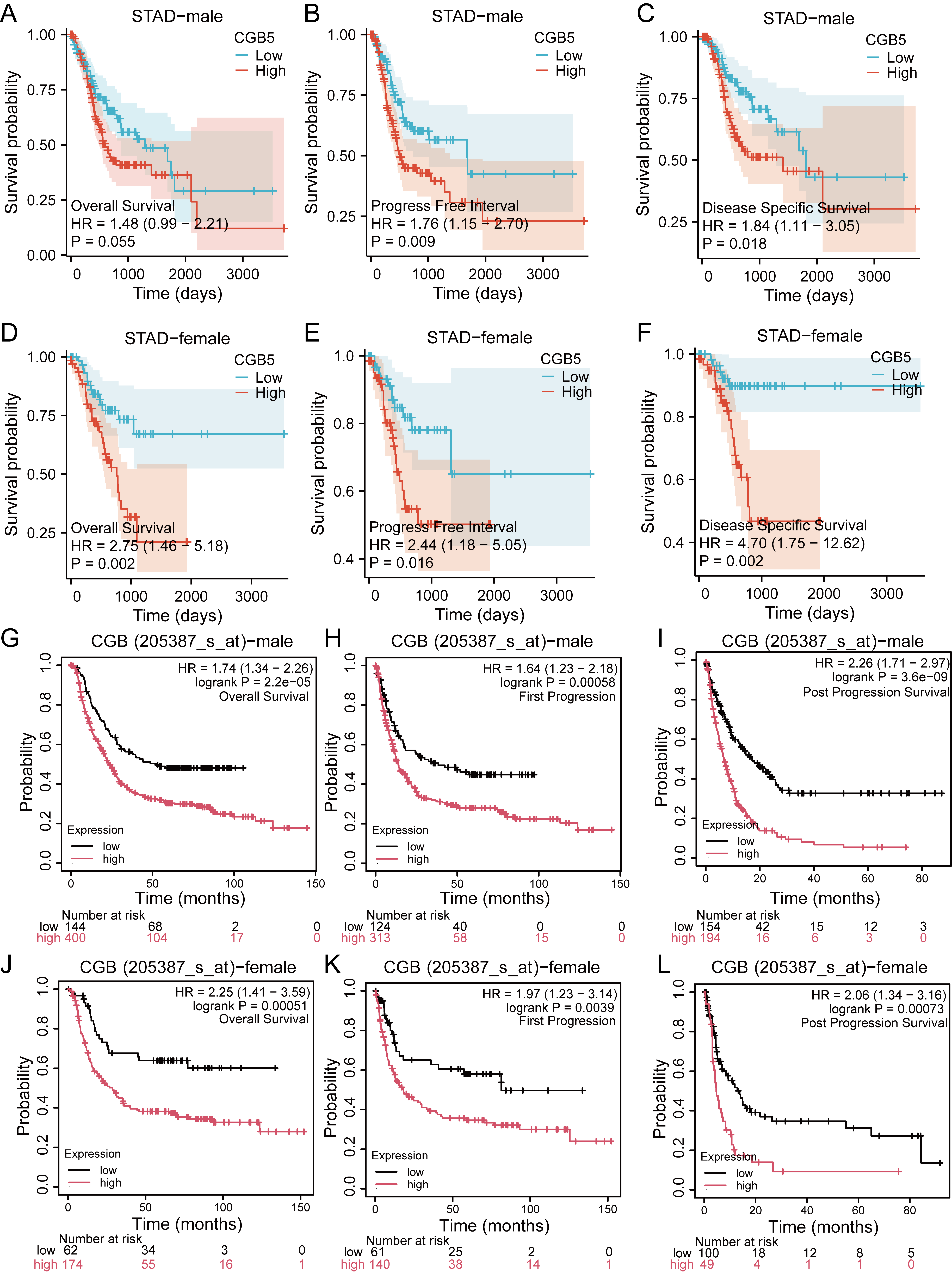
**

**Figure S7. Subgroup analysis of CGB5 in PAAD prognosis by gender.(A-C)**The impact of CGB5 expression on OS,DSS,PFI of male PAAD patients in TCGA database.**(D-F)**The impact of CGB5 expression on OS,DSS,PFI of female PAAD patients in TCGA database.(G-H)The impact of CGB5 expression on OS,DFS of male PAAD patients in GEO database.(I-J)The impact of CGB5 expression on OS,DFS of female PAAD patients in GEO database.

**
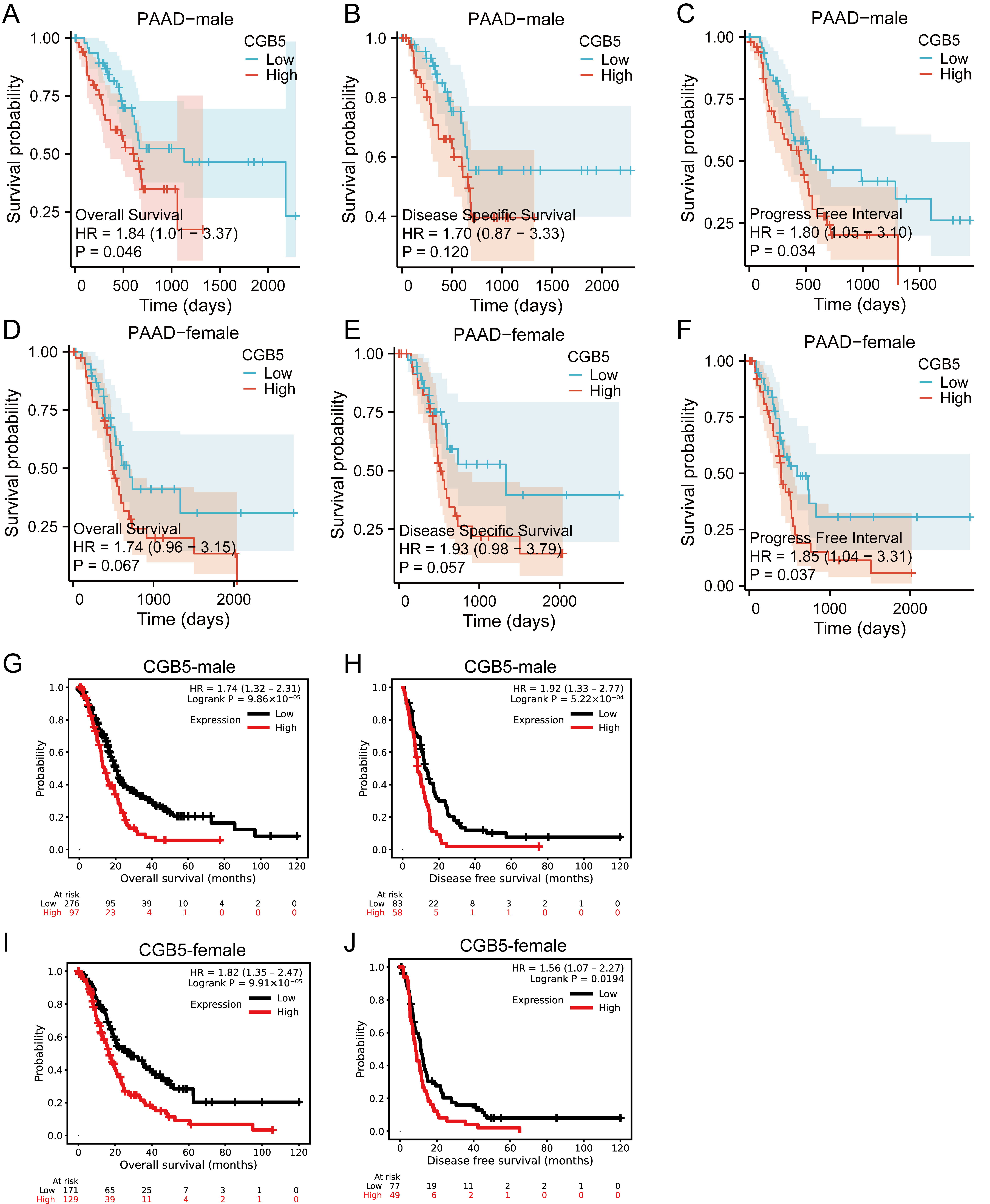
**

**Figure S8. Prognostic Significance of CGA Expression on OS in Pan-Cancer Analysis**

**
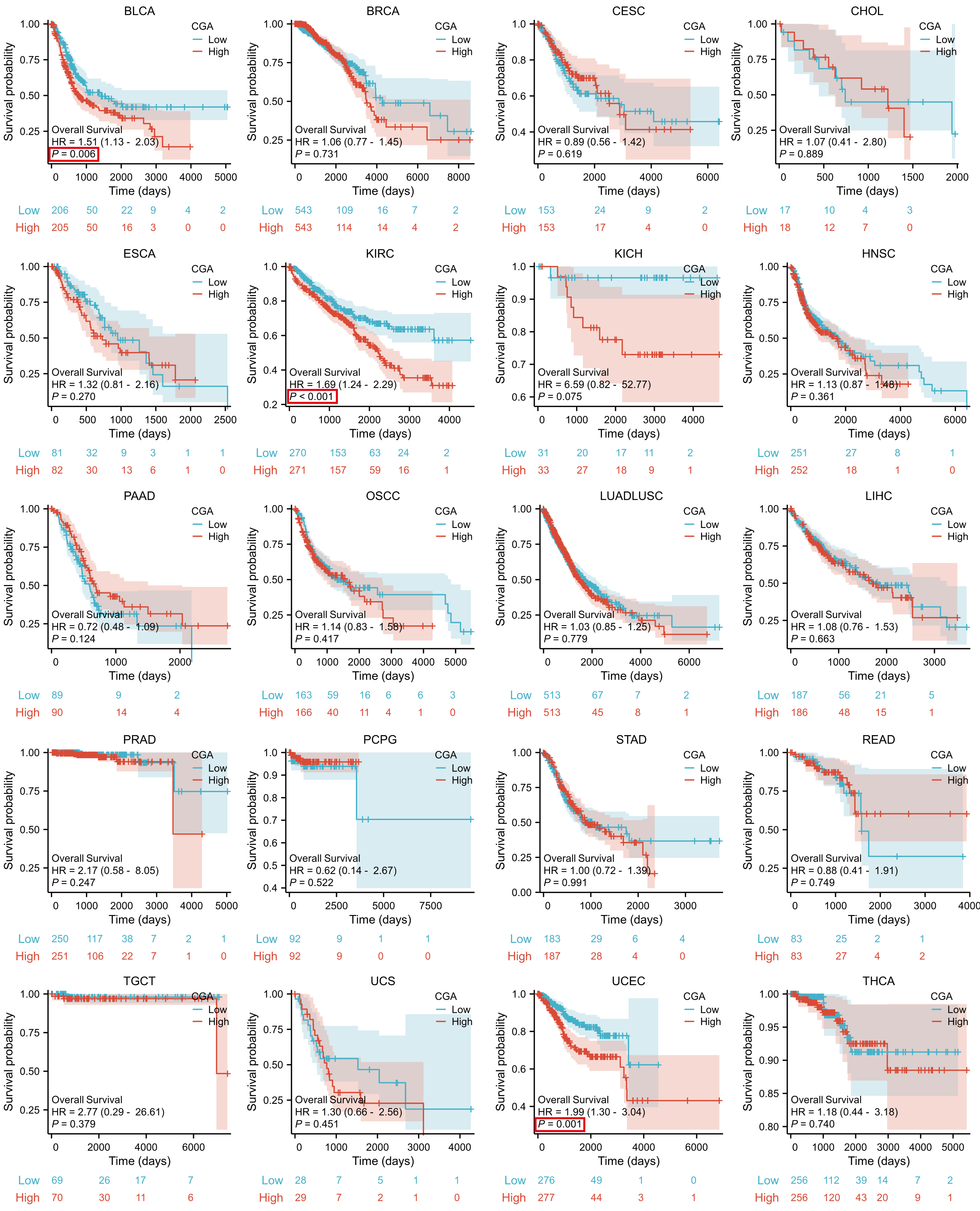
**

**Figure S9.** **Association between Immune Cell Infiltration and CGB5 Expression across Pan-Cancer.** The interaction between CGB5 expression and the infiltration levels of B cells, CAFs, and CD8+ T cells is visualized using the TIMER2.0 database. Positive correlations (ranging from 0 to 1) are indicated by the color red, whereas negative correlations (ranging from -1 to 0) are represented by the color blue. Statistically significant associations are defined as those with a *p*-value less than 0.05.


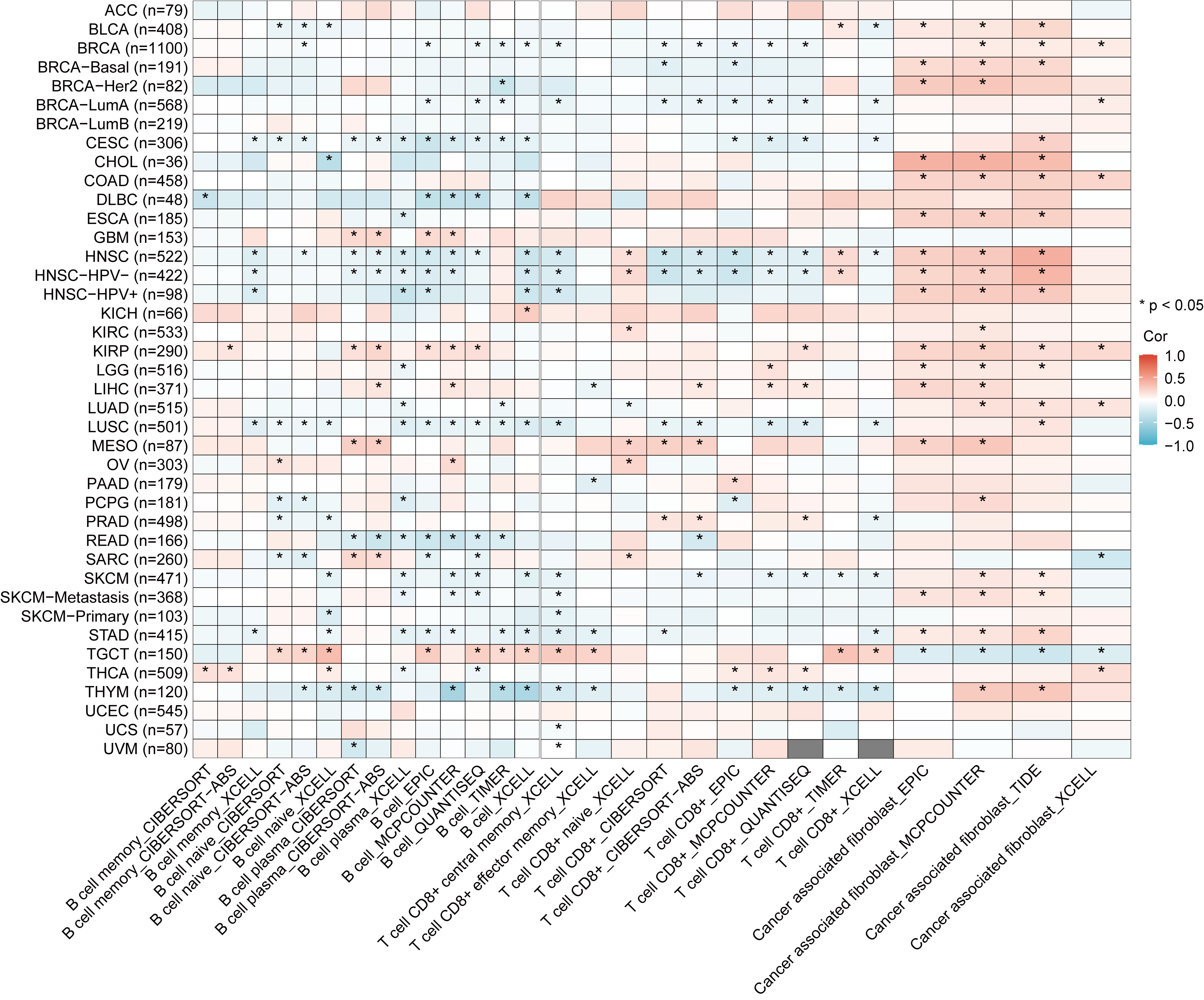


**Figure S10. Immune Infiltration Analysis of CGB5 in Pan-Cancer Using Various Algorithms**. (A) EPIC, (B) IPS, (C) MCPcounter, (D) TIMER, and (E) xCell.


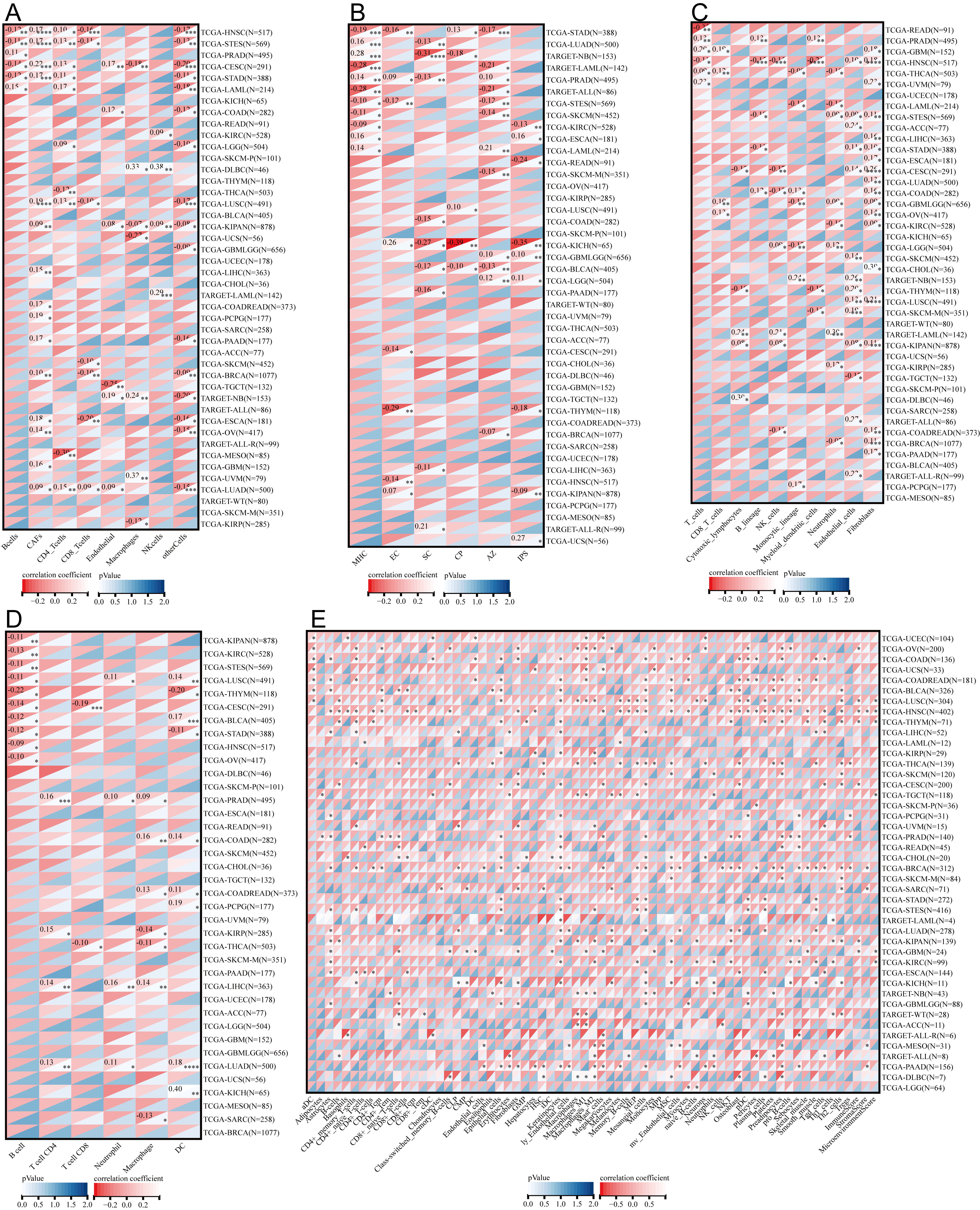


**Figure S11. Immune infiltration of CGB5 in pan-cancer with algorithms.** (A) Quantiseq, (B) Cibersort.


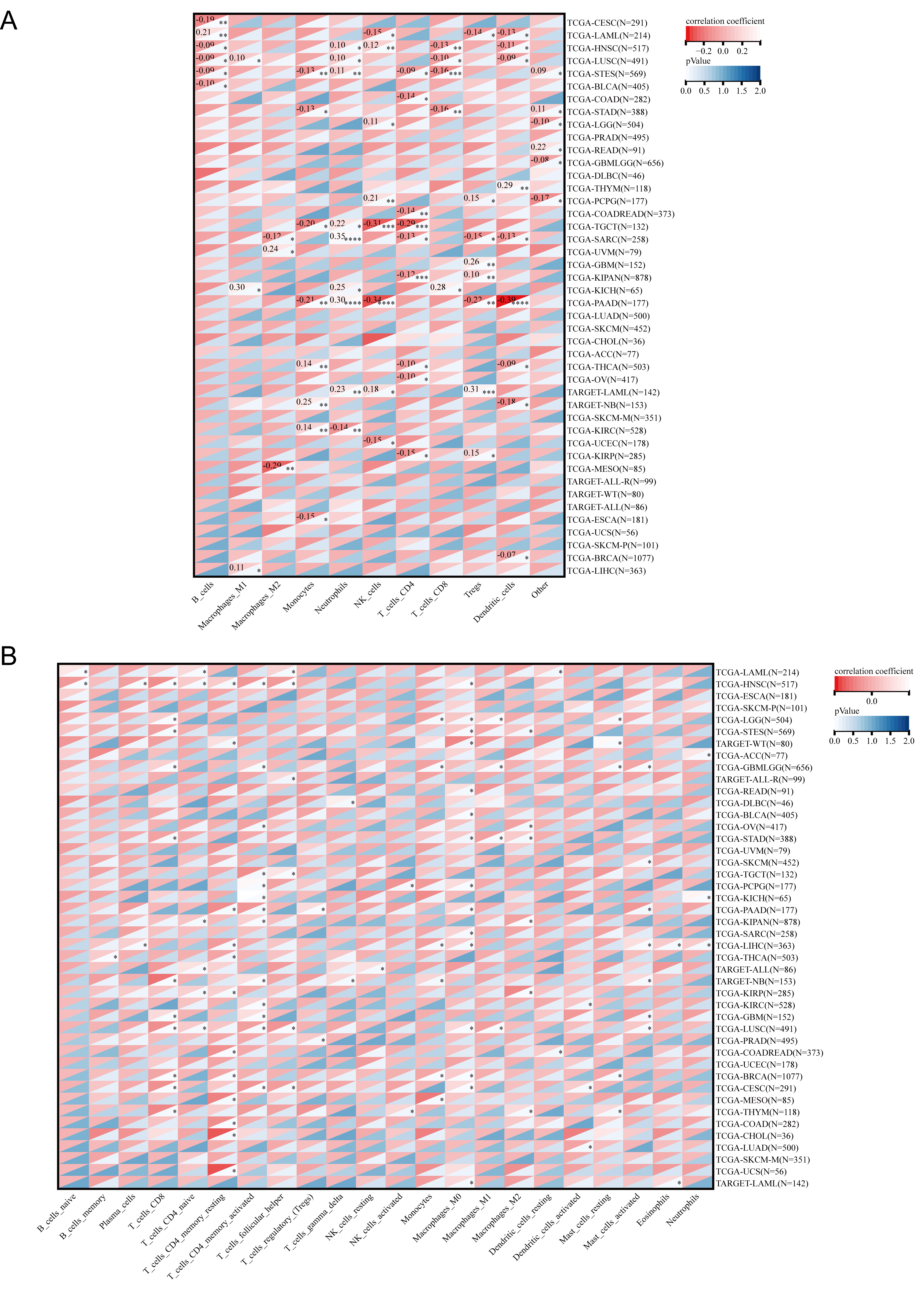


**Figure S12. The relationship between CGB5 expression and immune-related genes in pan-cancer.** (A) immunomodulatory genes, (B) immune checkpoint genes.


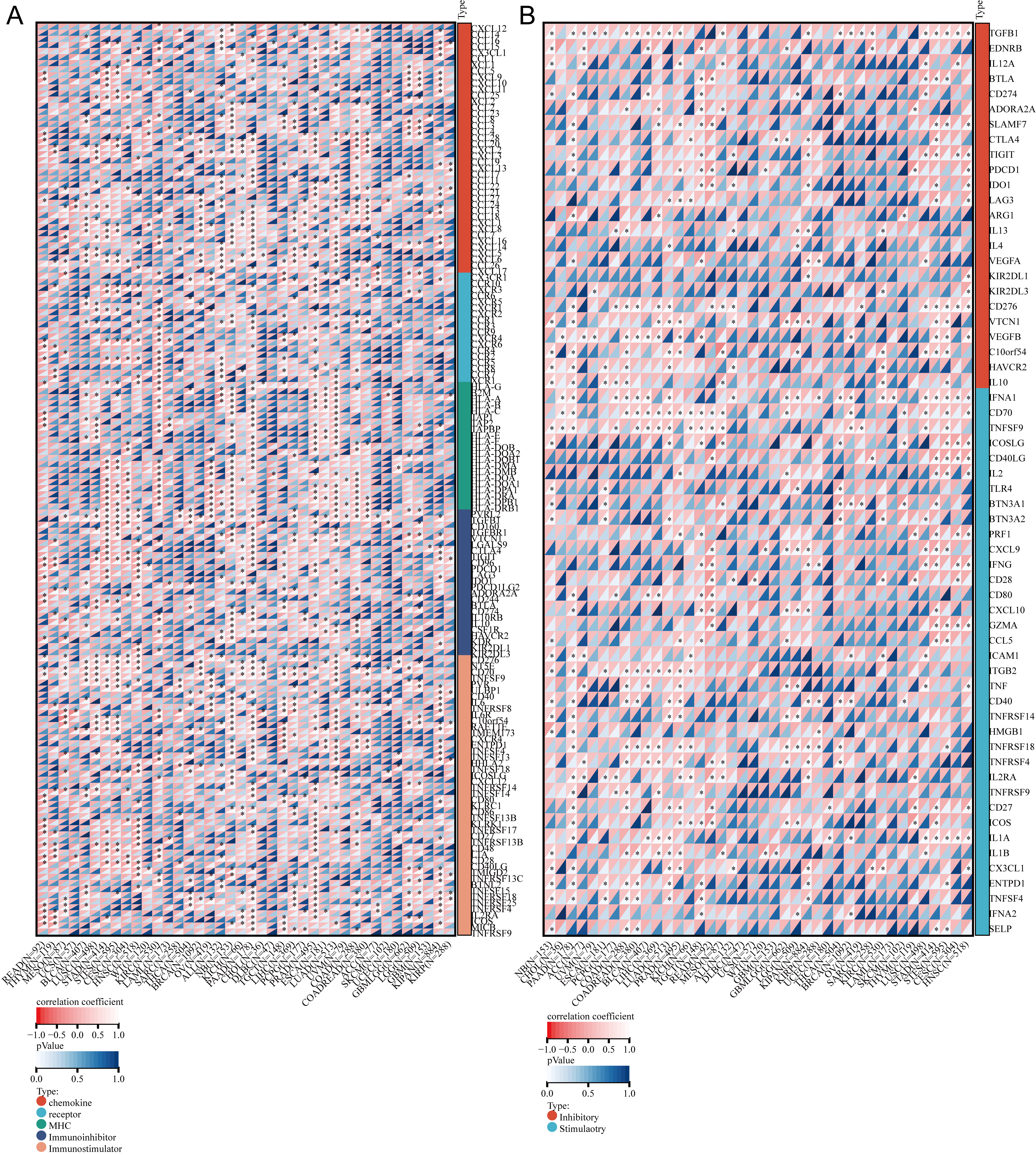


**Figure S13. Subgroup survival analyses of CGB5 in gastric cancer.** (A). DSS, (B). PFI.


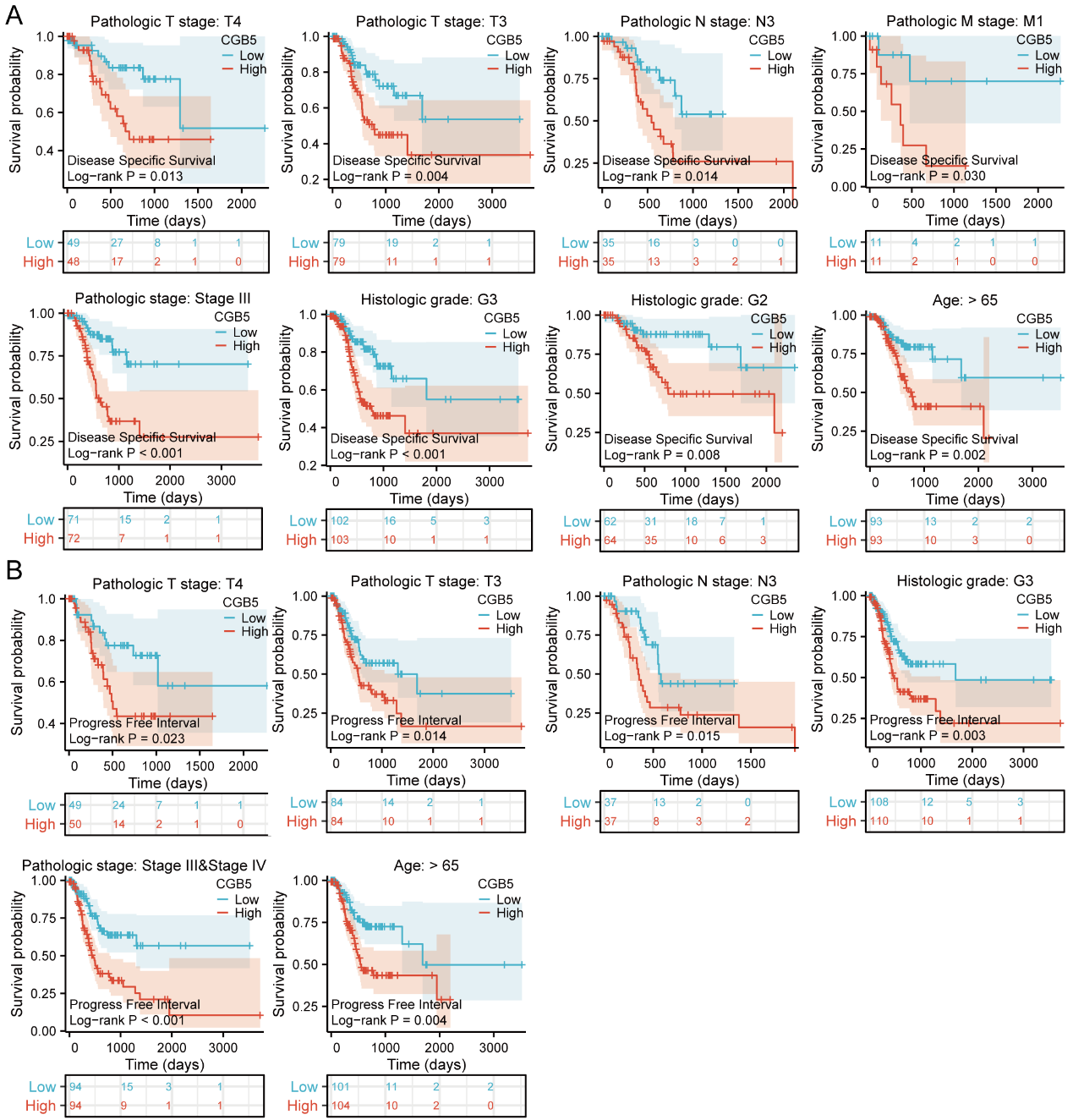

Supplement: Supplementary file 1 [file Data_Sheet_1.doc]
