# Supplementary material for: Comprehensive pan-cancer analysis reveals CGB5 is a potential promising predictive and immunotherapeutic biomarker
Source: Front Med (Lausanne). 2025 Sep 19;12:1624815. doi: 10.3389/fmed.2025.1624815 (PMC12491329; doi:10.3389/fmed.2025.1624815)
Supplement: Supplementary file 2 [file Table_1.DOCX]

The data involved in this study are described under the following categories:

1.1 Expression Data:

* TCGA and GTEx Data:

Data Acquisition: RNA-seq data (STAR-processed, TPM format) for the TCGA-STAD (Stomach Adenocarcinoma) project were downloaded and curated from the TCGA database (https://portal.gdc.cancer.gov). Additionally, uniformly processed TCGA and GTEx RNA-seq data in TPM format were obtained from UCSC Xena . Data corresponding to gastric adenocarcinoma from TCGA and relevant normal tissue data from GTEx were extracted.

Filtering Strategy: No specific filtering was applied. It is noted that statistical analyses were contingent upon meeting the following data characteristics per group: a minimum sample size of 3 and non-zero variance. Groups failing these criteria were excluded from subsequent statistical tests.

Processing Method: log2(value + 1) transformation was applied.

1.2 Survival Data:

* TCGA Data:

Data Acquisition: RNA-seq data (STAR-processed, TPM format) and corresponding clinical data for the TCGA-STAD (Stomach Adenocarcinoma) project were downloaded and curated from the TCGA database (https://portal.gdc.cancer.gov).

Supplementary Data: Prognostic information was supplemented using data from a published Cell study (PMID: 29625055).

Filtering Strategy: Normal samples and samples lacking associated clinical information were removed.

Processing Method: log2(value + 1) transformation was applied to expression values.

* GEO Data:

The prognostic significance of CGB5 in gastric cancer was assessed using the Kaplan-Meier Plotter tool (https://kmplot.com/analysis/). Filtering criteria applied during the analysis adhered to the data usage guidelines provided on the Kaplan-Meier Plotter website.

**Analysis Using UALCAN Platform**

Analyses performed through the UALCAN web interface utilize the UALCAN pre-processed and normalized expression data. UALCAN applies its own normalization pipeline to TCGA data, which is designed for comparisons within and across cancer types on its platform.

**TIMER Database Analysis for Immune Infiltration**

Immune cell infiltration estimates from TIMER were generated using the algorithm provided by the TIMER2.0 web server. The TIMER method incorporates specific normalization steps and signature gene sets designed to account for platform variation and tissue background.

**TISIDB Database Analysis for Immune and Subtype Associations**

Correlation analyses between CGB5 and immune markers in TISIDB utilize the pre-calculated, batch-adjusted (using ComBat) correlation coefficients provided by the database for TCGA pan-cancer data.
